# NLR receptors in plant immunity: making sense of the alphabet soup

**DOI:** 10.15252/embr.202357495

**Published:** 2023-08-21

**Authors:** Mauricio P Contreras, Daniel Lüdke, Hsuan Pai, AmirAli Toghani, Sophien Kamoun

**Affiliations:** ^1^ The Sainsbury Laboratory University of East Anglia Norwich UK

**Keywords:** bioengineering, immunology, NB‐LRR, NBS‐LRR, NOD, Immunology, Microbiology, Virology & Host Pathogen Interaction, Signal Transduction

## Abstract

Plants coordinately use cell‐surface and intracellular immune receptors to perceive pathogens and mount an immune response. Intracellular events of pathogen recognition are largely mediated by immune receptors of the nucleotide binding and leucine rich‐repeat (NLR) classes. Upon pathogen perception, NLRs trigger a potent broad‐spectrum immune reaction, usually accompanied by a form of programmed cell death termed the hypersensitive response. Some plant NLRs act as multifunctional singleton receptors which combine pathogen detection and immune signaling. However, NLRs can also function in higher order pairs and networks of functionally specialized interconnected receptors. In this article, we cover the basic aspects of plant NLR biology with an emphasis on NLR networks. We highlight some of the recent advances in NLR structure, function, and activation and discuss emerging topics such as modulator NLRs, pathogen suppression of NLRs, and NLR bioengineering. Multi‐disciplinary approaches are required to disentangle how these NLR immune receptor pairs and networks function and evolve. Answering these questions holds the potential to deepen our understanding of the plant immune system and unlock a new era of disease resistance breeding.

## Graphical abstract

Pathogen recognition in plants is largely mediated by immune receptors of the nucleotide binding and leucine rich‐repeat (NLR) classes. This review covers basic aspects of NLR biology and networks and highlights recent advances in NLR structure, function, and activation.

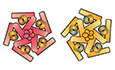

## Introduction

Plants have a sophisticated, multi‐layered innate immune system that actively protects them against pathogen invasion (Jones *et al*, 2016; Ngou *et al*, 2022a). They coordinately use cell‐surface and intracellular immune receptors to perceive pathogens and mount an immune response. To successfully infect their host, pathogens secrete virulence proteins, termed effectors. Effectors typically promote disease by modulating host physiology and suppressing cell‐surface or intracellular immunity (Couto & Zipfel, 2016; Wu & Derevnina, 2023). Some of these effectors are recognized by the protein products of plant *resistance (R)* genes, which largely belong to the nucleotide binding and leucine rich‐repeat (NLR) classes (Kourelis & Van Der Hoorn, 2018). Effector recognition by NLRs leads to effector‐triggered immunity (ETI), also known as NLR‐triggered immunity. ETI is a robust immune response usually accompanied by a form of programmed cell death known as the hypersensitive response or hypersensitive cell death (Jones & Dangl, 2006; Ngou *et al*, 2022a).

In the 1950s, Harold Flor first proposed a framework for host‐pathogen interactions termed the gene‐for‐gene model, in which matching pairs of genes from a host and a pathogen determine the outcome of a given interaction (Flor, 1971). Many NLR‐effector pairs follow this model, where the presence of a single pathogen gene, termed the avirulence (AVR) gene, triggers immunity in hosts carrying a single matching NLR gene (Jones & Dangl, 2006). These AVR genes usually encode effector proteins. As such, plant parasites are under constant pressure from their hosts to diversify their effector gene repertoire to evade recognition while maintaining virulence. On the other hand, plants and their NLRs constantly evolve their resistance gene repertoire to keep up with rapidly evolving pathogens. This has resulted in tremendous genetic innovation, with NLR‐coding genes being the most diverse genes in plants (Clark *et al*, 2007; Baggs *et al*, 2017; Barragan & Weigel, 2021). Over time, this evolutionary arms race has likely contributed to an increase in NLR complexity, with NLRs becoming sub‐functionalized and evolving from single individual genetic units, or “singletons,” to higher order configurations, such as NLR pairs or networks.

In NLR pairs and networks, multiple immune receptors work together to achieve robust immunity. Sensor NLRs mediate pathogen perception and activate downstream helper NLRs, which mediate immune signaling. Unlike NLR pairs, which function in one‐to‐one sensor–helper connections, NLR networks simultaneously exhibit many‐to‐one and one‐to‐many functional sensor–helper connections, likely contributing to increased robustness and evolvability of the plant immune system (Wu *et al*, 2017, 2018; Adachi *et al*, 2019b; Feehan *et al*, 2020).

In this review, we aim to cover the basic aspects of plant NLR biology, with an emphasis on NLR network biology. We touch on recent breakthroughs in NLR structure, function, and activation, which have provided insights into how NLRs translate pathogen perception into immune signaling via oligomerization‐based mechanisms. Moreover, we discuss emerging areas of study in plant NLR biology such as modulator NLRs, pathogen suppression of NLRs, and NLR bioengineering. Overall, we highlight the wide structural and functional diversity of NLR‐mediated immunity along with the higher order of complexity presented by NLR pairs and networks.

### What is an NLR?

NLR proteins are found across all kingdoms of life and exhibit a conserved tripartite modular domain architecture (Uehling *et al*, 2017; Duxbury *et al*, 2021; Gao *et al*, 2022; Kibby *et al*, 2023). In their broadest definition, they are STAND (signal transduction ATPases with numerous domains) proteins comprised of an N‐terminal domain, a central nucleotide‐binding and oligomerization domain (NOD), and C‐terminal superstructure‐forming repeats (SSFRs; Dyrka *et al*, 2020; Kourelis *et al*, 2021). N‐terminal domains are usually thought of as signaling domains, and in plant NLRs, they often mediate the downstream programmed cell death response following immune receptor activation (Duxbury *et al*, 2021). The plant NLR NOD is exclusively an NB‐ARC (nucleotide‐binding adaptor shared by APAF‐1, certain *R* gene products, and CED‐4) domain, whereas the C‐terminal SSFRs are typically leucine‐rich repeat (LRR) domains (Kourelis *et al*, 2021).

Plant NLRs, like most STAND proteins, are molecular switches (Takken *et al*, 2006). They exist in an inactive ADP‐bound resting state and conditionally initiate immune signaling upon perception of nonself or modified‐self (Takken & Goverse, 2012; Jones *et al*, 2016; Gao *et al*, 2022). The central NB‐ARC domain is critical for mediating conformational changes required for switching between inactive and active states, primarily through the exchange of ADP for ATP at its nucleotide binding pocket (Takken *et al*, 2006; Wang *et al*, 2019b). While the C‐terminal LRR can, in some cases, determine pathogen perception, this domain also mediates critical autoinhibitory intramolecular interactions that hold the receptor in an inactive state prior to activation (Takken & Goverse, 2012; Förderer *et al*, 2022). Following activation and release of intramolecular auto‐inhibition, the N‐terminal domains can subsequently mediate downstream immune signaling.

Plant NLRs exhibit diverse N‐terminal signaling domains, which can be used to broadly classify them into distinct classes. These classes follow the phylogeny of the NB‐ARC domain, indicating that they have a deep evolutionary origin (Kourelis *et al*, 2021). To date, four main N‐terminal signaling domains have been characterized in angiosperms: Coiled‐coil (CC)‐type, RESISTANCE TO POWDERY MILDEW 8 (RPW8)‐type (CC_R_), G10‐type CC (CC_G10_), and toll/interleukin‐1 receptor‐type (TIR; Kourelis *et al*, 2021). NLRs in nonflowering plants can carry additional types of N‐terminal domains, such as α/β hydrolases and kinase domains (Andolfo *et al*, 2019; preprint: Chia *et al*, 2022). In general, the N‐terminal domain is thought to dictate the types of downstream signaling pathways and activities that take place following NLR activation.

Although the majority of plant NLRs retain the canonical and presumably ancestral tri‐partite domain organization, many NLRs have diversified into specialized proteins with additional noncanonical domains or degenerated features (Cesari *et al*, 2014; Adachi *et al*, 2019a; Kourelis *et al*, 2021). As we uncover more NLR structural and functional diversity, it is imperative that we revise the way in which we conceptualize NLR domains and their roles in immune receptor activities.

### NLRs are highly expanded and diverse in plants

In addition to their role in plant defense, NLRs have also been the subject of extensive investigation in the field of evolutionary biology, as they have undergone rapid evolution and diversification in response to strong selection pressures from rapidly evolving pathogens. Large‐scale comparative phylogenomic analyses have revealed that NLR‐encoding genes are some of the most diverse and quickly evolving in plant genomes (Clark *et al*, 2007; Barragan & Weigel, 2021; Prigozhin & Krasileva, 2021). They occur in all major groups of flowering plants (angiosperms) and nonflowering plants, with some NLR‐like genes being found in green algae (Andolfo *et al*, 2019; Shao *et al*, 2019; preprint: Chia *et al*, 2022). This phylogenetically informed view of NLRs revealed that they are diverse in many ways. The number of NLRs varies greatly across species, ranging from ~50 in watermelon (*Citrullus lanatus*) and papaya (*Carica papaya*) to > 1,000 in apple (*Malus domestica*) and hexaploid wheat (*Triticum aestivum*; Jia *et al*, 2015; Baggs *et al*, 2017; Steuernagel *et al*, 2020). NLRs exhibit lineage‐specific expansions and contractions, which usually occur through tandem duplication and/or deletion events in each species often influenced by transposon content, ecological context, and adaptation to their environment (Baggs *et al*, 2017; Barragan & Weigel, 2021). NLR genes also exhibit tremendous intraspecific diversity, exhibiting presence/absence variation and heterogeneity in allelic variation, largely due to point mutations, intra‐allelic recombination, and domain fusions or swaps (Seeholzer *et al*, 2010; MacQueen *et al*, 2019; Maekawa *et al*, 2019; van de Weyer *et al*, 2019; Seong *et al*, 2020; Prigozhin & Krasileva, 2021; preprint: Lin *et al*, 2022; Shimizu *et al*, 2022).

The recently generated RefPlantNLR collection of almost 500 experimentally validated NLRs nicely illustrates our current grasp of NLR diversity in terms of domain architecture and function, showcasing that plants have evolved NLRs to detect effectors from most plant pathogenic organisms (Kourelis *et al*, 2021). Importantly, looking at the plant species represented in the RefPlantNLR dataset highlighted that most NLRs characterized to date come from a relatively small pool of flowering plant species. Our understanding of broader NLR domain structure and molecular function, in particular outside of crop and model plant species or in nonflowering plants, is therefore limited. Only recently, a preprinted study by Chia *et al* (preprint: Chia *et al*, 2022) functionally characterized NLRs and NLR signaling domains from deep lineages of land plants and algae, revealing that there are indeed shared NLR activities spanning the whole spectrum of plant evolution. Notably, this study leveraged transient heterologous expression in the model flowering plant *Nicotiana benthamiana* as a powerful tool to perform functional screens of N‐terminal CC, CC_R,_ and TIR‐type signaling domains from divergent algal and plant genomes. They found that many of these retained the capacity to trigger hypersensitive cell death like their angiosperm counterparts. This indicates that some NLR signaling domains and their functions arose early during plant evolution and have retained these functions over a long evolutionary time (preprint: Chia *et al*, 2022). Nonetheless, much NLR functional diversity in underrepresented or understudied plant species remains to be explored. This study is a perfect example of how powerful these comparative approaches can be in identifying important commonalities and differences in NLR function. Integrating phylogenetics, evolutionary biology, and functional studies into NLR research is critical to uncovering the full potential of these diverse plant proteins.

### NLR receptors are intracellular sensors of invading pathogens

Plant NLRs sense intracellular effectors delivered by pathogens during infection and subsequently trigger an immune response (Jones & Dangl, 2006; Lolle *et al*, 2020). The strategies by which NLRs recognize effectors can generally be divided into two categories: direct and indirect recognition. Direct recognition of effectors follows a receptor‐ligand model, with one NLR protein binding one effector molecule (Baggs *et al*, 2017). For example, the wheat CC‐NLR Sr35 directly binds the effector AvrSr35 via its LRR domain. Effector binding relieves intramolecular autoinhibition in Sr35 and triggers conformational rearrangements that lead to Sr35 activation (Förderer *et al*, 2022; Zhao *et al*, 2022). The TIR‐NLRs RPP1 from *Arabidopsis* and Roq1 from *N. benthamiana* also recognize their cognate effectors via direct binding. RPP1 recognizes the effector ATR1 from the oomycete *Hyaloperonospora arabidopsidis*, while Roq1 recognizes the *Xanthomonas perforans* effector XopQ. In both cases, effector binding occurs in the LRR, assisted by a post‐LRR region found in some TIR‐NLRs known as the C‐terminal jelly roll and Ig‐like domain (C‐JID). Effector binding by RPP1 and Roq1 also induces conformational rearrangements leading to NLR activation and downstream signaling (Ma *et al*, 2020; Martin *et al*, 2020).

Sr35, RPP1, and Roq1 are well‐studied cases in which cryogenic electron microscopy (Cryo‐EM) structures of NLRs in complex with their cognate effectors have resolved the ligand binding interfaces with intricate detail, but many additional examples of NLRs that directly recognize effectors exist (Jia *et al*, 2000; Dodds *et al*, 2006; Catanzariti *et al*, 2010; Chen *et al*, 2017; Zhu *et al*, 2017; Bauer *et al*, 2021). In most of these examples, the LRR domain plays a critical role in determining effector recognition specificity. For the tomato CC‐NLR Sw5‐b, which directly recognizes the NSm viral protein of different tospoviruses (Peiró *et al*, 2014; Zhu *et al*, 2017), the LRR also plays a key role in effector binding, although an additional domain located before the N‐terminal CC domain, known as the Solanaceous domain (SD), also contributes to direct interaction with the effector (Zhu *et al*, 2017).

While direct recognition of effectors by NLRs would seem like the most intuitive and simple strategy to perceive pathogens, there are comparatively more examples in which effectors are indirectly recognized. Some NLRs can monitor or “guard” host components targeted by pathogen effectors, which are therefore termed “guardees” (Jones & Dangl, 2006). The CC‐NLR Prf from tomato guards the host kinase Pto by sensing its interaction with the bacterial effectors AvrPto and AvrPtoB to trigger Prf‐dependent immune signaling (Kim *et al*, 2002). The RPS5 CC_G10_‐NLR from *Arabidopsis* guards the host kinase PBS1. Cleavage of PBS1 by the bacterial protease AvrPphB leads to RPS5‐mediated immunity (Ade *et al*, 2007). In their attempts to manipulate host physiology and immunity, different effectors, either from the same, or phylogenetically unrelated pathogens, sometimes converge on the same host proteins to promote disease (Song *et al*, 2009; Mukhtar *et al*, 2011; Macho & Zipfel, 2015; Derevnina *et al*, 2021; Petre *et al*, 2021). One example is the recognition of the *P. syringae* effectors AvrRpm1, AvrB, and AvrRpt2. These three effectors modify the host protein RIN4, phosphorylating it in the case of AvrRpm1 and AvrB or cleaving it in the case of AvrRpt2. In turn, the CC‐NLR RPM1 and the CC_G10_‐NLR RPS2 guard RIN4, sensing its phosphorylation or cleavage, respectively, leading to immunity (Axtell & Staskawicz, 2003; Mackey *et al*, 2003).

**Figure 1:**
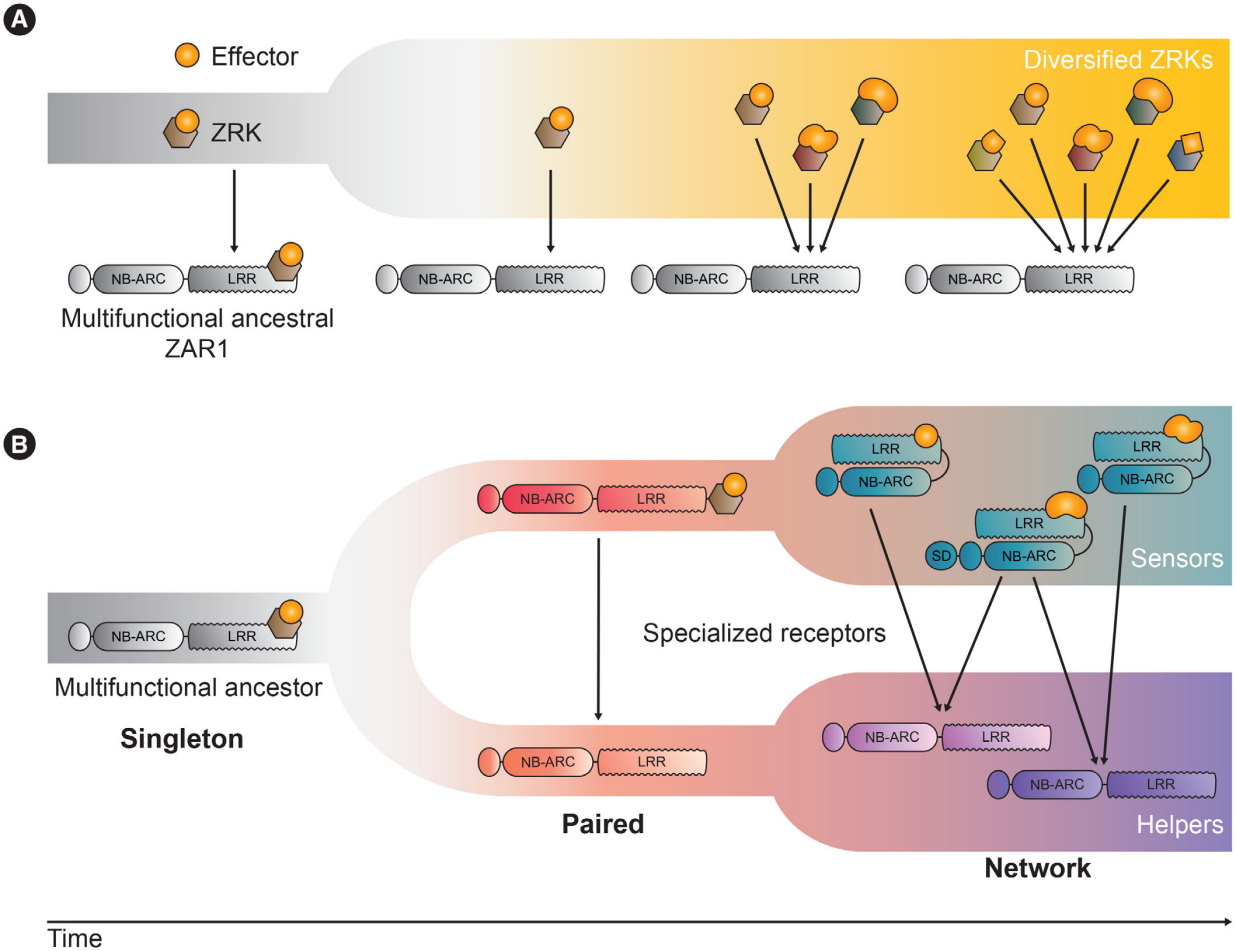
Evolution of NLR singletons, pairs, and networks (A) The NLR ZAR1 indirectly recognizes multiple bacterial effectors by guarding host RLCKs, also termed ZRKs, which bait bacterial effectors. Over evolutionary time, ZRKs have greatly diversified as a result of coevolution with pathogen effectors. In contrast, ZAR1 has remained atypically conserved throughout angiosperm evolution, relying on ZRKs for pathogen recognition and specializing in interacting with ZRKs to mediate immune signaling. (B) NLRs can be categorized into functional singletons, pairs, and networks. While singletons can mediate both pathogen sensing and downstream immune signaling, NLRs have duplicated and diversified over evolutionary time, leading to the appearance of specialized receptors that can be defined as either “sensors” or “helpers,” forming connections that range from pairs to complex networks.

A derivation of the guard‐guardee model is the decoy model. Whereas some NLR guardees are functional host proteins with discernible physiological roles, decoys are host proteins that evolved to bait pathogen effectors without other clear functions in host physiology (van der Hoorn & Kamoun, 2008). For example, ZAR1 can recognize a range of bacterial effectors through its partner receptor‐like cytoplasmic kinases (RLCKs), also termed ZED1‐related kinases (ZRKs; Fig 1A; Wang *et al*, 2015; Seto *et al*, 2017; Schultink *et al*, 2019; Laflamme *et al*, 2020; Adachi *et al*, 2023b). ZED1 and RKS1 are two such RLCKs that constitutively form a complex with ZAR1. The *Xanthomonas campestris pv. campestris* effector HopZ1a acetylates ZED1, and this modification is sensed by ZAR1, triggering its activation. The effector AvrAC from *P. syringae* uridylylates the RLCK PBL2. This modified PBL2 then conditionally interacts with the preformed ZAR1‐RKS1 complex, triggering an immune response. Because ZED1 is a pseudokinase and PBL2 uridylylation does not enhance AvrAC‐mediated virulence, these host proteins are considered decoys dedicated to baiting pathogen effectors (Lewis *et al*, 2013; Wang *et al*, 2015).

How does indirect recognition aid in keeping up with rapidly evolving pathogen effectors? Indirect effector recognition allows plants to maximize the efficacy of a fixed number of immune receptors. NLRs that indirectly recognize pathogens by guarding common virulence targets are more versatile than direct effector binders, as they hold the potential to recognize multiple effectors simultaneously, even if these effectors are unrelated in structure or sequence or are secreted by different pathogens and pests. From an evolutionary point of view, guarding pathways that are commonly targeted by multiple pathogens enables plants to have a wider surveillance mechanism than responding to specific pathogen molecules. Moreover, guardees and decoys that are dedicated to effector sensing should be less evolutionarily constrained than NLRs that execute the immune response. This model has analogies to the NLR sensor/helper pairing discussed below, except that the sensing activity is performed by a non‐NLR moiety. Along these lines, guardees and decoys can potentially accumulate a higher number of mutations because they do not retain the original functionality of their progenitor protein allowing them to better keep up with rapidly evolving effectors (Fig 1A and B).

### NLR‐IDs: NLRs with unconventional integrated domains

Moving beyond the canonical tri‐partite domain architecture, up to 10% of the NLRome of a given plant species can consist of NLRs with additional insertions known as integrated domains (IDs). These IDs often mimic the host targets of effectors (Cesari *et al*, 2014; Kroj *et al*, 2016; Sarris *et al*, 2016). Within the NLR, IDs can be involved in effector sensing, either via direct or indirect recognition (Fujisaki *et al*, 2015; Maqbool *et al*, 2015; De la Concepcion *et al*, 2018; Gu *et al*, 2023). The integrated decoy hypothesis postulates that over evolutionary time, host targets of effectors have genetically integrated within NLRs, baiting pathogen effectors to activate host immunity (Cesari *et al*, 2014). The well characterized rice CC‐NLRs Pik‐1 and RGA5 (aka Pia‐2) feature an integrated domain known as Heavy Metal Associated (HMA), which binds multiple effectors from the rice blast fungus, *Magnaporthe oryzae*, to mediate disease resistance. These NLRs can directly recognize multiple *M. oryzae* effectors: Pik‐1 recognizes AVR‐Pik and AVR‐Mgk1, while RGA5 recognizes AVR1‐CO39 and AVR‐Pia (Cesari *et al*, 2013; Maqbool *et al*, 2015; De la Concepcion *et al*, 2018; Guo *et al*, 2018; Sugihara *et al*, 2023).

Identification of the host targets of effectors recognized by NLR‐IDs supported the hypothesis that IDs are derived from host target integration. For both Pik‐1 and RGA5, the cognate effectors are sequence unrelated but share a conserved structural fold, termed the MAX fold (Magnaporthe AVRs and ToxB‐like; de Guillen *et al*, 2015). MAX fold effectors have been shown to bind endogenous “non‐integrated” HMA proteins from the host, supporting the notion that over evolutionary time HMAs have integrated into NLRs to mimic the host targets of MAX effectors (preprint: Oikawa *et al*, 2020; Bentham *et al*, 2021; Białas *et al*, 2021; Maidment *et al*, 2021). Another well‐studied example is the RRS1 TIR‐NLR from *Arabidopsis*, which features a C‐terminal integrated WRKY transcription factor‐like domain. The bacterial effectors AvrRps4 from *P. syringae* and PopP2 from *Ralstonia solanacearum* can modify host WRKY transcription factors to promote disease (Pandey & Somssich, 2009; Le Roux *et al*, 2015; Sarris *et al*, 2015). The C‐terminal integrated WRKY domain of RRS1 acts as a bait for these effectors, as RRS1 can sense its modification to activate immunity (Le Roux *et al*, 2015; Mukhi *et al*, 2021). Interestingly, all functionally characterized NLR‐IDs to date require a second, genetically linked, NLR to confer disease resistance and are therefore known as “paired” NLRs (Cesari *et al*, 2014; Adachi *et al*, 2019b).

A diversity of domains have fused with NLRs over evolutionary time, suggesting that there is a degree of flexibility in terms of what domains can potentially be integrated into NLR‐IDs (Sarris *et al*, 2016; Marchal *et al*, 2022a). A striking case is the Pia/Pias allelic/ortholog series from the *Oryza* genus. In this example, variants of the same NLR exhibit at least nine different integrations, including HMA domains, protein‐kinase domains, or WRKY domains (Shimizu *et al*, 2022). While recent works have shed light on how IDs evolve following integration and how intramolecular ID‐NLR scaffold interactions shape NLR‐ID function (Białas *et al*, 2021; De la Concepcion *et al*, 2021), how NLRs evolve to generate novel domains is not fully understood (Box 1). Understanding NLR‐ID evolution can inform NLR bioengineering strategies, as discussed in the corresponding section of this review.

Box 1 **In need of answers**

1. How do additional domains become integrated into the canonical NLR domain structure? How do these integrated domains (IDs) evolve following integration? How do they co‐evolve with their cognate effectors and their NLR chassis? Understanding how NLR‐IDs evolve and function in nature may enable the bioengineering of novel made‐to‐order disease resistance genes.
2. How do NLR pairs and networks evolve? How do they activate, and how are they regulated? What are the molecular determinants for sensor–helper compatibility within NLR networks? Elucidating the molecular basis of sensor–helper specificity could inform bioengineering efforts to make the plant immune system more robust against pathogen perturbation.
3. What are the precise mechanisms by which activated CC‐NLR and CC_R_‐NLR resistosomes mediate immune signaling and disease resistance? While their calcium channel activity is required for cell death, the precise downstream mechanisms by which NLR resistosomes trigger cell death are not understood.
4. What is the molecular link between cell‐surface and intracellular immune receptors? Understanding the interplay, synergies and dependencies of cell‐surface and intracellular immunity is key to achieving a more holistic view of the plant immune system.
5. What are the molecular determinants of sub‐cellular NLR localization pre‐ and post‐activation? What is the nature of the membrane‐associated puncta formed by NLR resistosomes?
6. To what degree do plants use NLRs to respond to pathogens in a cell type‐ or tissue‐specific manner? How does NLR signaling function in different plant tissues? Are there cell‐autonomous and non‐cell‐autonomous NLR responses?
7. How prevalent are NLR suppressors? What is their precise contribution to pathogen virulence? NLR‐suppressing effectors may be masking a vast and yet untapped reservoir of disease resistance genes in crop genomes. A better understanding of how pathogens suppress NLR signaling may enable new disease resistance strategies.

### NLR singletons and pairs

Some NLRs function as individual genetic units and are therefore termed functional singleton NLRs. These can directly or indirectly perceive pathogen effectors and induce a downstream immune response without relying on additional NLRs (Fig 1A and B; Adachi *et al*, 2019a). Well‐studied immune receptors in this category include the CC‐NLRs ZAR1 from *Arabidopsis* and Sr35 from wheat. As mentioned previously, ZAR1 indirectly recognizes its cognate effectors via its guardee and decoy RLCKs, while Sr35 binds AvrSr35 directly via its LRR (Wang *et al*, 2019a; Förderer *et al*, 2022; Zhao *et al*, 2022). Other NLRs that are considered likely singletons based on their capacity to sense effectors and trigger hypersensitive cell death in heterologous plant systems include Sr50, several NLRs in the MLA allelic series, RPS5, and RPP13 (Qi *et al*, 2012; Ravensdale *et al*, 2012; Chen *et al*, 2017; Maekawa *et al*, 2019; Saur *et al*, 2019). To this end, *Agrobacterium tumefaciens*‐mediated transient expression (aka agroinfiltration) of NLR proteins in the leaves of *N. benthamiana* has been a useful method for heterologous expression of immune receptors. If a CC‐NLR from an unrelated species is capable of triggering cell death in *N. benthamiana*, it will likely function as a singleton or helper. Although this test is not definitive and depends on the degree of conservation of potential downstream signaling partners, decoys, or guardees, it serves as a first approach to classifying NLRs into functional categories and further exemplifies how *N. benthamiana* can be an excellent system to quickly functionally characterize and categorize NLRs (Derevnina *et al*, 2019; Adachi *et al*, 2019b).

**Figure 2:**
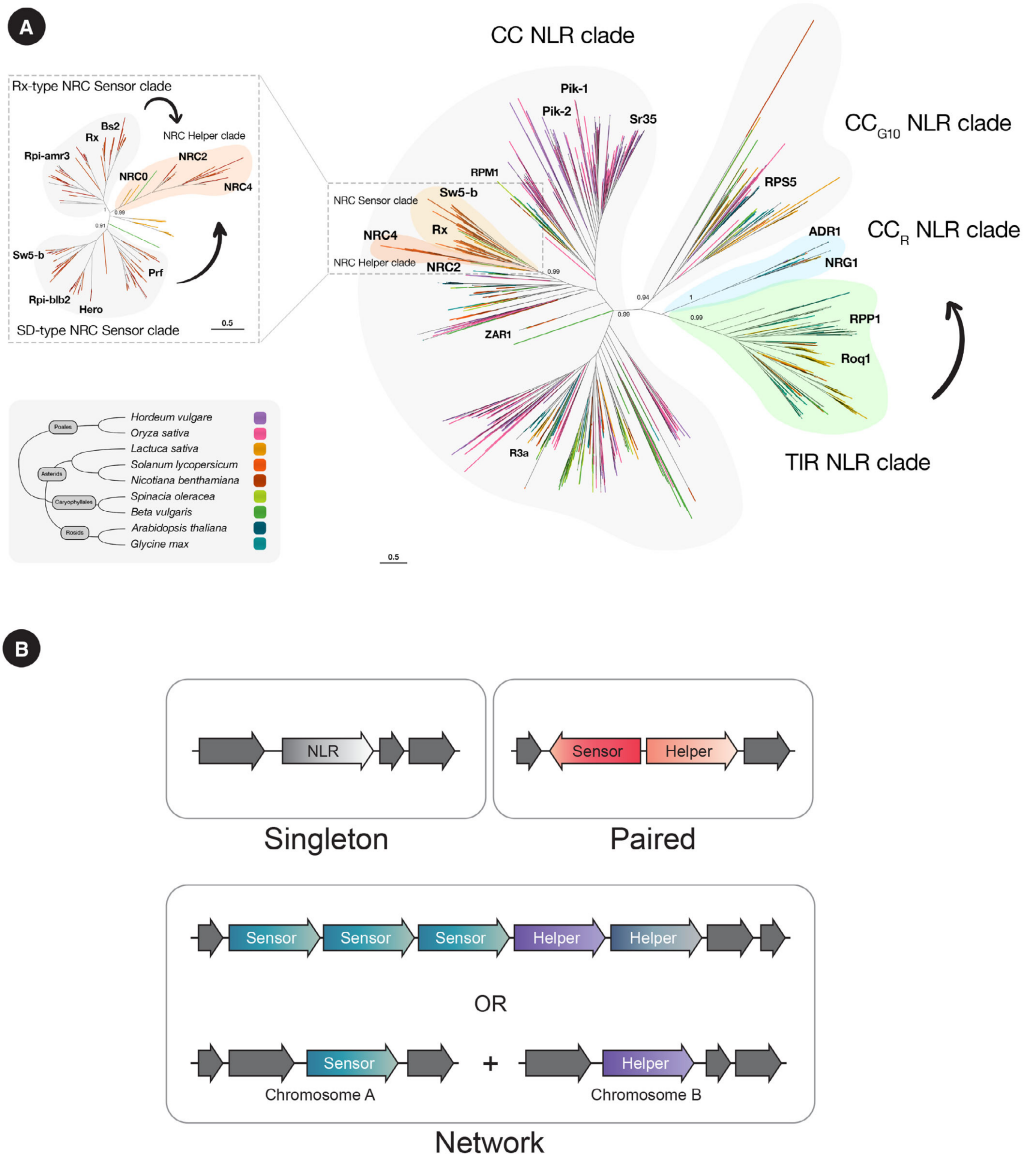
Phylogenetics of NLR networks (A) NLR networks exhibit a distinct phylogenetic structure, with sensors and helpers belonging to well‐supported clades. In the NRC super‐clade network, NRC‐sensors (light orange) group into an expanded clade, whereas NRC‐helpers (dark orange) form a tighter sister clade. The CC_R_ NLR clade helpers (blue), which are paired with TIR‐NLRs (green) sensors, also belong to well‐supported clades. The Phylogenetic tree is based on the NB‐ARC domain extracted from the NLRome of nine selected species representing poales, asterids, caryophyllales, and rosids. The phylogenetic relationship of NLRs was inferred by an approximately maximum‐likelihood model using FastTree. The branches of the NLR tree are colored according to species, as indicated in the species overview tree. CC_G10_, CC_R_, TIR NLR, and CC NLR (including NRC sensors and helpers) clades are outlined, and respective bootstrap values for each main branch are provided. Arrows indicate functional connections between clades. The position of the CC_R_ and TIR NLR as neighboring clades does not necessarily reflect a common phylogenetic origin and can be due to the divergence of the other deep branches in the NLR phylogeny. (B) While paired NLR sensors and helpers, derived from duplication and diversification, tend to be genetically linked, the sensors and helpers of NLR networks can be genetically dispersed.

In paired NLR systems, one immune receptor is specialized in pathogen perception acting as a sensor and requires a downstream helper NLR to induce immune signaling (Fig 1B). Some well‐studied “model” paired NLR systems include the rice Pik‐1/Pik‐2 and RGA5/RGA4 (Pia) CC‐NLR pairs and the *Arabidopsis* RRS1/RPS4 TIR‐NLR pair. The genes coding for the Pik‐1 NLR‐ID and its helper NLR Pik‐2 are found in head‐to‐head orientation and are both required for resistance to the blast fungus *M. oryzae* (Fig 2B). Pik‐1 binding to *M. oryzae* effectors leads to activation of immunity via its helper Pik‐2, with both NLRs working cooperatively to mediate disease resistance. Pik‐1 is unable to trigger cell death in the absence of its downstream helper Pik‐2 (Maqbool *et al*, 2015; Zdrzałek *et al*, 2020; De la Concepcion *et al*, 2021). In the case of RGA5 and its helper RGA4, these NLRs work by negative regulation rather than by cooperation (Césari *et al*, 2014). The RGA4 helper has been shown to be constitutively active, triggering cell death when heterologously expressed in *N. benthamiana* (Césari *et al*, 2014). Co‐expression of its sensor NLR‐ID mate RGA5 can suppress this constitutive activity. Upon effector binding by RGA5, this negative regulation is relieved enabling RGA4 to mediate immune signaling (Césari *et al*, 2014).

**Figure 3:**
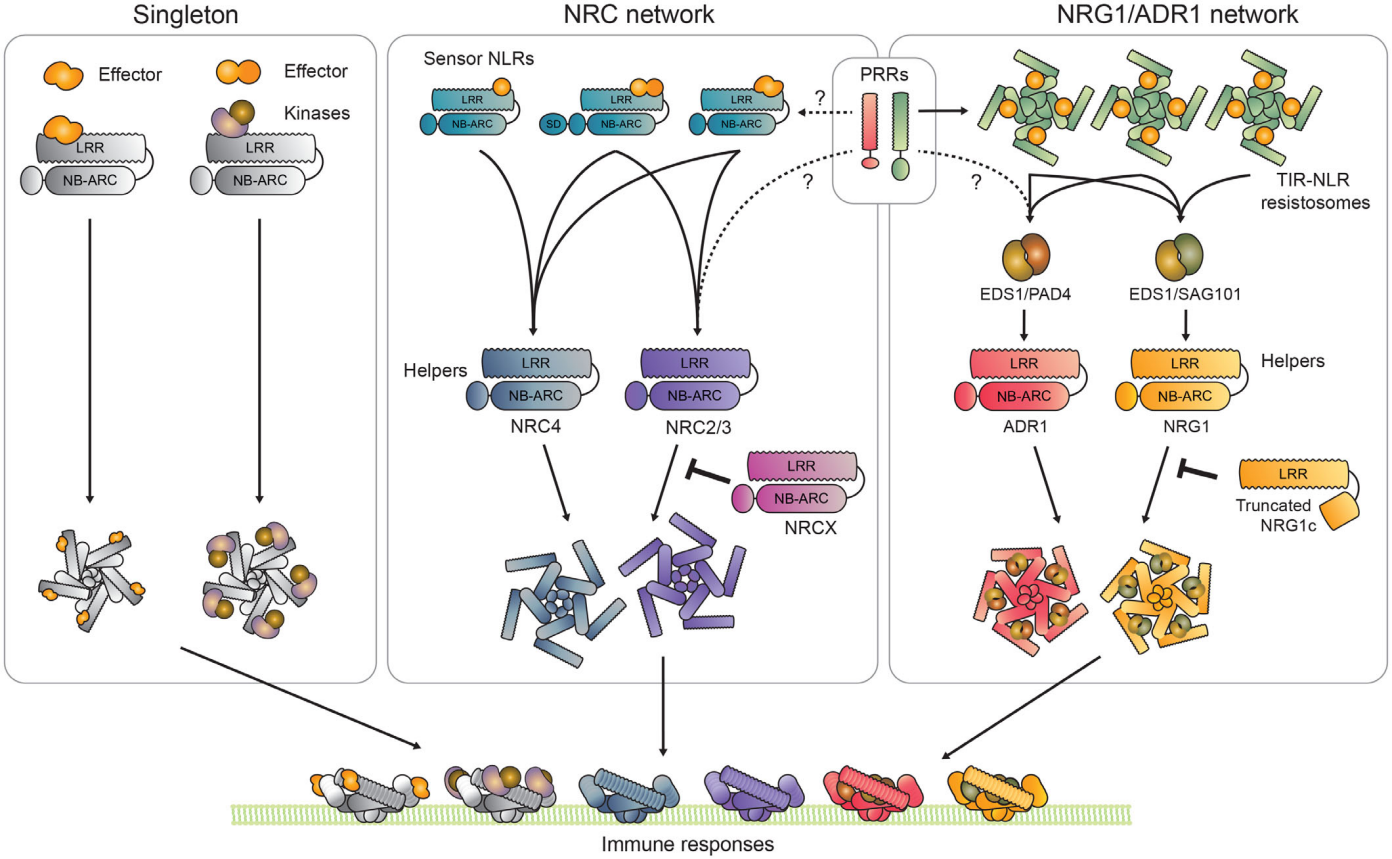
NLR activation mechanisms *Singleton*: Upon direct or indirect effector perception, singleton CC‐NLRs such as ZAR1 and Sr35 activate by forming homo pentameric complexes termed resistosomes. Resistosomes accumulate at the plasma membrane to initiate immune signaling and mediate programmed hypersensitive cell death, presumably by acting as calcium channels. *NRC network*: The NRC PRR/NLR network employs an activation‐and‐release mechanism. Sensory NLRs activate upon perceiving their cognate effectors and relay an unknown signal to their downstream helper NRCs, leading to their oligomerization. These resistosome‐like NRC homo‐oligomers accumulate at the plasma membrane, separate from the sensors that activated them. The NRC network also features atypical modulator NLRs like NRCx, which can regulate the signaling of the NRC helpers NRC2 and NRC3. The functional specialization into sensors and helpers greatly enhances immune receptor evolvability. For example, some sensor NLRs have acquired N‐terminal SDs involved in pathogen sensing. Although PRRs such as Cf4 and Ve1 have been shown to require NRC3 for cell death induction, whether they activate helpers directly or whether there is a sensor NLR mediating PRR‐helper NLR communication is not known (indicated by “?”). *NRG1/ADR1 network*: TIR‐NLR singletons and pairs form tetrameric resistosomes upon activation. These resistosomes act as holoenzymes, producing a range of small molecules that are perceived by downstream lipase‐like protein dimers EDS1‐PAD4 and EDS1‐SAG101. These activated EDS1‐PAD4 and EDS1‐SAG101 complexes can interact with the helper CC_R_‐NLRs ADR1 and NRG1, respectively, leading to their oligomerization into CCR‐NLR resistosomes. Following their activation and oligomerization, the ADR1 and NRG1 resistosomes accumulate on the PM to act as calcium‐permeable channels, leading to immune signaling and hypersensitive cell death. This network also features an atypical modulator NLR, NRG1c, which can negatively regulate immune signaling by full‐length NRG1. TIR‐NLRs, such as the CSA1/CHS3 pair, can act downstream of PRRs. The EDS1/PAD4/ADR1 node, and, to a lesser degree, the EDS1/SAG101/NRG1 node have also been shown to act downstream of various PRRs. In most cases, how PRRs signal to these nodes is not fully understood (indicated by “?”).

The genetically linked *Arabidopsis* TIR‐NLR pair RRS1/RPS4 similarly works via negative regulation. RPS4 is constitutively active in *Arabidopsis*, triggering immune signaling in the absence of pathogen infection if the RRS1 sensor, which acts as its repressor, is not present. RRS1 inhibition of RPS4 is conditionally relieved upon effector perception. While RRS1 and RPS4 are genetically linked paired NLRs, they require a genetically unlinked downstream helper CC_R_‐NLR, NRG1, to confer disease resistance (Figs 1B and 3). Therefore, whether RPS4 should be termed a helper for RRS1 is up for debate, as the term helper is often associated with a CC or CC_R_‐type NLR acting downstream of a sensor (Gong *et al*, 2023). These, as well as additional examples of paired NLR systems have been extensively reviewed (Adachi *et al*, 2019b; Feehan *et al*, 2020; Xi *et al*, 2022; Marchal *et al*, 2022a; Gong *et al*, 2023).

### NLR networks: the emergence of complex NLR systems

In some cases, NLRs have evolved more complex connections beyond paired sensor–helper configurations. Cases in which more than two NLRs are connected functionally are referred to as NLR networks (Wu *et al*, 2017, 2018; Duxbury *et al*, 2021; Adachi & Kamoun, 2022; Kourelis & Adachi, 2022). Networked NLRs can be genetically unlinked yet phylogenetically related and exhibit a sensor and helper dynamic (Figs 1B and 2A). Unlike paired NLRs, which exhibit one‐to‐one connections, NLR network components can exhibit “one‐to‐many” and “many‐to‐one” signaling architectures. Different sensors can converge on one downstream helper, and each individual sensor can signal redundantly via more than one helper (Wu *et al*, 2017, 2018). Helpers themselves are not necessarily fully redundant and can exhibit a degree of functional specialization, both in terms of compatibility with upstream sensors and downstream signaling (Wu *et al*, 2017; Saile *et al*, 2020). NLR networks combine the higher degree of receptor evolvability conferred by sensor–helper specialization, as seen in paired NLR systems, with the robustness conferred by genetic redundancy at the helper level (Figs 1B and 3; Wu *et al*, 2017; Castel *et al*, 2019; Adachi *et al*, 2019b; Gong *et al*, 2023).

In Solanaceous plants, helper NLRs known as NRCs (NLR Required for Cell death) are genetically required for immune signaling by a multitude of sensor NLRs and cell‐surface receptors that mediate perception of diverse plant pathogenic oomycetes, fungi, nematodes, insects, viruses, and bacteria (Wu *et al*, 2017; Derevnina *et al*, 2021; Kourelis *et al*, 2022; Oh *et al*, 2023). In *Nicotiana benthamiana*, the CC‐NLRs NRC2, NRC3, and NRC4 act as key nodes in this network (Wu *et al*, 2017). Another well‐characterized example is the NRG1/ADR1 network, in which the CC_R_‐NLRs NRG1 and ADR1 act as helpers, inducing immune signaling and cell death downstream of multiple TIR‐type sensor NLRs (TIR‐NLRs; Bonardi *et al*, 2011; Qi *et al*, 2018; Castel *et al*, 2019; Wu *et al*, 2019).

The prevalence of NLR pairs and networks in plants indicates the limits of the conceptual framework of NLR domains and their functions, which describes the canonical NLR functions in the context of a functional singleton. For example, in the case of helper NLRs that do not engage in effector recognition, the LRR may have adopted new activities, such as mediating sensor–helper communication.

### Asymmetrical evolution of paired and networked NLRs

In some cases, notably the NRC helpers and their sensors, CC‐NLR pairs and networks have been proposed to originate from multifunctional “singleton” ancestors that have biochemically subfunctionalized over evolutionary time (Wu *et al*, 2018; Adachi *et al*, 2019b; Fig 1B). This division of labor presumably facilitates keeping up with rapidly evolving pathogen effectors as it results in reduced evolutionary constraints for both the sensor and helper, thus enhancing the evolvability of the network components and the system as a whole. By specializing, NLR pairs broaden the potential spectrum of amino acid changes and domain integrations that can be accommodated in each of the immune receptors, since individual sensor and helper proteins no longer need to fulfill both functions (Adachi *et al*, 2019a). The acquisition of new domains for pathogen detection in sensor NLRs is likely facilitated by this functional specialization. For example, the majority of characterized NLR‐IDs to date are paired with helper NLRs (Marchal *et al*, 2022a). In the Pik and Pia paired NLR allelic series, higher levels of variation are found in the sensor NLR, specifically within the effector‐sensing ID (Białas *et al*, 2021; De la Concepcion *et al*, 2021; Shimizu *et al*, 2022). In the NRC network, many NRC‐dependent sensor NLRs include the N‐terminal Solanaceous Domain (SD), which can participate in pathogen perception (Mucyn *et al*, 2006; Lukasik‐Shreepaathy *et al*, 2012; Li *et al*, 2019). That NLRs with additional pathogen sensing domains such as IDs or SDs are found in paired or networked configurations therefore highlights the enhanced evolvability likely granted by sensor–helper NLR specialization (Figs 1B and 3; Wu *et al*, 2017; Marchal *et al*, 2022a). It is also conceivable that the evolutionary steps leading to the incorporation of additional domains would be less likely to lead to autoimmunity in a functionally specialized sensor NLR than in a singleton.

Paired or networked signaling configurations can also lead to the regressive evolution of NLR proteins. The MADA motif found in the N‐terminal ⍺1‐helices of around 20% of all angiosperm CC‐NLRs is crucial for cell death initiation. MADA and MADA‐like motifs, such as the MAEPL motif of nonflowering plants, are present and functionally conserved in evolutionarily distant NLRs that can execute cell death. Examples of NLRs with an N‐terminal MADA motif include singleton NLRs such as the atypically ancient NLR ZAR1, helper NLRs such as Pik‐2 and NRC4, and several *Marchantia polymorpha* CC‐NLRs, indicating that these CC‐NLRs share a common origin and that the MADA‐type ⍺1‐helix traces back to the origin of land plants (Adachi *et al*, 2019a,b; preprint: Chia *et al*, 2022). In contrast, sensor NLR specialization toward pathogen perception is often accompanied by loss or degeneration of the MADA motif, making these proteins incapable of activating immunity on their own (Adachi *et al*, 2019a).

In the Pik‐1/Pik‐2 NLR pair, the Pik‐2 helper carries an N‐terminal MADA motif, whereas the Pik‐1 sensor does not (Adachi *et al*, 2019a). Pik‐1 features an integrated HMA domain through which it interacts with pathogen effectors and is unable to trigger cell death in the absence of Pik‐2 (Kanzaki *et al*, 2012; Zdrzałek *et al*, 2020; De la Concepcion *et al*, 2021). Similarly, while the NRC helpers contain a MADA motif, none of the NRC‐dependent sensors carry a detectable MADA sequence (Adachi *et al*, 2019a; preprint: Chia *et al*, 2022). Moreover, the N‐termini of MADA or MAEPL motif containing NLRs can functionally substitute the N‐terminus of the helper NRC4, whereas the N‐termini of sensor CC‐NLRs cannot (Adachi *et al*, 2019a; preprint: Chia *et al*, 2022). This evolutionary “use‐it‐or‐lose‐it” model postulates that conserved motifs in the N‐termini of sensor NLRs degenerated into “non‐functional” sequences as a consequence of relying on helpers for cell death induction (Adachi *et al*, 2019a,b).

### Phylogenomics of NLR pairs and networks: making sense of the alphabet soup

The genomic location of NLR genes across plant species has revealed clues about their evolutionary history. NLRs can be encoded in genetic clusters or dispersed as genetic singletons. In *Arabidopsis*, around 50% of all NLRs are found in clustered arrangements that arose from tandem gene duplication or unequal crossing‐over events (van de Weyer *et al*, 2019; Lee & Chae, 2020). NLRs that are dispersed throughout the genome often lack introns and were proposed to have expanded through retroduplication events (Kim *et al*, 2017). These gene duplication events enable NLRs to sub‐functionalize, acquire new activities and domains, and functionally diverge, leading to a variety of gene clusters that consist of either a series of phylogenetically related genes or NLR genes of mixed origin (Adachi *et al*, 2019b).

NLR sensor–helper pairs, such as rice Pik‐1/Pik‐2 or Pia (aka RGA5/RGA4), are usually genetically clustered in tight physical linkage, sometimes in head‐to‐head orientation (Césari *et al*, 2014; Stein *et al*, 2018; Białas *et al*, 2021; Fig 2B). This presumably allows for transcriptional co‐regulation of sensors and helper genes. The Pik‐1 and Pik‐2 pairs, for example, are transcribed from a shared promoter (Ashikawa *et al*, 2008; De la Concepcion *et al*, 2018). This physical linkage also facilitates the co‐segregation of functionally connected sensor and helper pairs. This is important considering that some helpers are constitutively active, as discussed above, and inheritance of the helper without the sensor would have deleterious effects on the plant.

In contrast, sensors and helpers in the NRC network of asterid plants are often genetically dispersed even though they have a common evolutionary origin and belong to defined phylogenetic clades (Wu *et al*, 2017; Fig 2A). For example, all NRC‐dependent sensors fall into an expanded clade that includes many well‐known resistance (R) proteins from different solanaceous plant species, while the NRC helpers form a tight and well‐supported sister clade. Wu *et al* (2017) proposed that this network dates back to ~100 million years ago and probably originated from a pair of sensor/helper genes, which itself likely evolved from a singleton (Figs 1B and 2A and B; Wu *et al*, 2017; Adachi *et al*, 2019b; Adachi & Kamoun, 2022).

Another well‐studied NLR network is the TIR‐NLR/CC_R_‐NLR network, where helper NLRs like NRG1 and ADR1 execute the hypersensitive response following pathogen activation of TIR‐NLR sensors. Remarkably, both TIR NLRs and CC_R_ NLRs form well‐supported phylogenetic clades, although they may not have a common evolutionary origin. It is tempting to hypothesize that this network evolved from the functional pairing of single TIR‐NLR and CC_R_‐NLR genes that are associated with additional signaling components (Liu *et al*, 2023).

In the future, the phylogenetic position of NLRs, combined with the presence or absence of functional sequence motifs, the occurrence of integrated domains, and other variables, should prove useful in categorizing NLRs into functional classes and revealing new pairs and networks from naive plant genomes (Adachi *et al*, 2019a,b). Indeed, one of the challenges posed by the rapid acceleration in sequencing plant genomes is making sense of their NLR alphabet soup. Ultimately, we will be able to integrate multiple parameters to identify NLR networks in any given plant species, determine their architecture, and subsequently validate predicted functional links between NLRs.

### Functional consequences of genetically dispersed NLR networks

Sensors and helpers in both the NRC network and the TIR‐NLR/CC_R_‐NLR network are genetically dispersed NLR genes, in contrast to the majority of paired NLRs. Does the lack of genetic linkage among networked NLRs confer an evolutionary advantage? It is possible that genetically unlinked NLR networks allow the generation of more regulatory diversity or for the acquisition of novel domains, such as the SD domain found in many NRC‐dependent sensors or the IDs found in multiple TIR‐NLRs (Figs 1B and 3; Seong *et al*, 2020; Kourelis *et al*, 2021). In these networks, which mediate recognition of multiple pathogens, the occurrence of the sensor genes across several genome locations might facilitate birth‐and‐death evolution, enabling modular gene loss when selection pressure against one pathogen is relieved without affecting resistance to other pathogens.

It is possible that helper NLR genetic redundancy combined with the convergence of multiple sensors into one downstream helper facilitates the loss of genetic linkage over evolutionary time, in contrast to paired NLRs where a sensor exclusively functions with a single helper. How these NLR networks became unlinked and how they have adapted to the lack of co‐regulation provided by genetic linkage is not understood. These genetically dispersed NLR networks add another layer of complexity to the evolutionary paradigm of the gene‐for‐gene model, as sensor NLRs (typically the *R* genes) are simultaneously co‐evolving with pathogen effectors as well as with their downstream helpers.

Despite the above considerations, genetically dispersed NLR networks can carry trade‐offs for the plant. For instance, NLR misregulation can result in autoimmunity, particularly when plants from different populations are crossed (Bomblies *et al*, 2007; Chae *et al*, 2014; Atanasov *et al*, 2018). This phenomenon, known as Dangerous Mix (DM), results in so‐called hybrid necrosis and can be due to a mismatch between genetically unlinked NLR gene loci. In one case, autoimmunity is triggered by a heterocomplex between two *Arabidopsis* TIR‐NLR proteins, DM1 (DM1^Uk‐3^) and DM2d (DM2d^Uk‐1^), that are encoded by genes on different chromosomes (Tran *et al*, 2017).

### Pathogen activation of NLR functional singletons

While the first plant NLR genes were cloned about 30 years ago (Bent *et al*, 1994; Whitham *et al*, 1994), the molecular mechanisms of NLR activation and immune signaling following pathogen perception were only elucidated recently. Using Cryo‐EM, Wang and colleagues obtained structural insights into the *Arabidopsis* singleton NLR ZAR1 before and after activation (Wang *et al*, 2019a,b). Following effector perception, ZAR1 undergoes a series of conformational changes largely mediated by the NB‐ARC domain (Wang *et al*, 2019b). This involves the exchange of ADP for ATP and ultimately leads to ZAR1 oligomerization and assembly into a pentameric wheel‐like homo‐oligomer, analogous to the mammalian inflammasome. This plant NLR oligomer was coined the resistosome (Hu *et al*, 2015, 2020; Zhang *et al*, 2015; Wang *et al*, 2019a,b; Xiao *et al*, 2023). NLR oligomerization leads to the induced proximity of the N‐terminal signaling domains. In the case of the CC‐NLR ZAR1, the α1‐helix of the CC domain containing the N‐terminal MADA‐motif flips out upon activation, forming a funnel‐like structure that mediates resistosome insertion into the plasma membrane (Fig 3; Wang *et al*, 2019a; Bi *et al*, 2021). Following insertion, the ZAR1 resistosome presumably acts as a calcium channel, an activity that is required for hypersensitive cell death (Bi *et al*, 2021). More recently, the ZAR1 oligomerization model has expanded to include additional MADA‐motif containing CC‐NLRs from distantly related plant species. The two functional singletons CC‐NLR Sr35 from wheat and RPP7 from *Arabidopsis* were shown to oligomerize into pentameric resistosome complexes, revealing a general mechanism of MADA‐CC‐NLR activations (Li *et al*, 2020; Förderer *et al*, 2022; Zhao *et al*, 2022).

### Pathogen activation in the NRC network

How does the resistosome activation model identified for singleton CC‐NLRs translate to CC‐NLR pairs or networks (Box 1)? Recent biochemical studies centered on NRC2 and NRC4 revealed that sensor–helper pairs in the NRC network employ an activation‐and‐release mechanism, in which effector perception by sensor NLRs induces oligomerization and resistosome formation of downstream helpers (Fig 3). The sensor does not appear to oligomerize or integrate into the helper resistosome (Ahn *et al*, 2023; Contreras *et al*, 2023b). NRC2 was shown to oligomerize when activated by multiple NRC2‐dependent sensor NLRs, including the *Potato virus X* (PVX) R protein Rx, the bacterial R protein Bs2, and the oomycete R proteins Rpi‐amr1 and Rpi‐amr3 (Ahn *et al*, 2023; Contreras *et al*, 2023b). The NRC2 oligomer is composed of multiple NRC2 units and has a size consistent with a pentameric complex. Following activation, NRC2 shifts from the cytoplasm to the plasma membrane, presumably to act as a calcium channel as previously described for other CC‐NLRs (Fig 3; Contreras *et al*, 2023b). The activation‐and‐release model contrasts with the activation mechanisms of metazoan‐paired NLRs, such as NAIP/NLRC4, which assemble into a heteromeric inflammasome upon activation, indicating that plant and metazoan NLR pairs and networks may exhibit distinct activation mechanisms (Hu *et al*, 2015; Vance, 2015; Contreras *et al*, 2023b).

The activation‐and‐release model further supports the notion that helper NLRs of the NRC network functionally specialize in immune signaling by retaining an intact N‐terminal MADA motif and oligomerizing into resistosomes (Adachi *et al*, 2019a,b). In contrast, NRC‐dependent sensors have diverged from the ancestral state by specializing in pathogen perception while no longer retaining the MADA motif or the capacity to oligomerize or form resistosomes (Fig 3).

NRC‐dependent sensors are not compatible with every NRC helper and exhibit a degree of specificity as illustrated by the network architecture (Wu *et al*, 2017; Derevnina *et al*, 2021; Contreras *et al*, 2023a,b). While some sensors, such as Rx, Bs2, and Rpi‐amr3 can signal indistinctly through NRC2, NRC3, or NRC4, other sensors exhibit a narrower helper specificity profile (Wu *et al*, 2017). For example, Rpi‐amr1 can signal through NRC2 but not NRC4, and conversely, Rpi‐blb2 can signal through NRC4 but not NRC2 (Wu *et al*, 2017; Duggan *et al*, 2021; Witek *et al*, 2021; Fig 3). Notably, these differential genetic dependencies could be recapitulated biochemically in the form of NRC helper oligomerization given that sensors can only oligomerize helpers they can productively signal through (Ahn *et al*, 2023; Contreras *et al*, 2023b; Fig 3). This supports the notion that networked immune receptor arrangements are more than mere genetic redundancies, as NRC helpers appear to exhibit a degree of functional specialization. The molecular determinants of sensor–helper compatibility, or lack thereof, are not understood. Elucidating the molecular basis of sensor–helper communication can yield new opportunities for engineering efficient NLR networks that confer more robust disease resistance.

The activation‐and‐release biochemical model for sensor–helper pairs in the NRC network has provided a new conceptual framework for networked CC‐NLR activation. NRC‐dependent sensors do not appear to form resistosomes and instead mediate downstream NRC activation and oligomerization upon pathogen perception. NRC helpers engage in resistosome formation and immune signaling, forming resistosomes that do not include the sensor (Ahn *et al*, 2023; Contreras *et al*, 2023b). However, how exactly activated sensor NLRs in the NRC network communicate with their downstream helpers for the formation of a helper resistosome remains an unsolved question. One hypothesis is that direct sensor–helper interactions trigger helper oligomerization via the formation of transient sensor–helper intermediate complexes. Alternatively, the sensors may be communicating with downstream helpers indirectly via unknown signaling partners or by producing signaling molecules (Box 1).

### NLR activation in the TIR‐NLRs/CC_R_‐NLR network

TIR‐NLRs form a receptor network with helper NLRs of the CC_R_ clade. This receptor network is phylogenetically structured in the sense that the sensors (TIR‐NLRs) and helpers (CC_R_‐NLRs) belong to well‐defined clades in the NLR tree (Figs 2A and 3). In this network, TIR‐NLRs are thought to rely on their CC_R_‐NLR helpers to cause hypersensitive cell death. The structures of the activated TIR‐NLRs Roq1 and RPP1 revealed that these distinct TIR‐NLRs also employ oligomerization‐based activation mechanisms. Following effector perception, both immune receptors form homo‐tetrameric resistosomes in which the TIR domains are brought together (Ma *et al*, 2020; Martin *et al*, 2020).

TIR‐NLR resistosomes function as holo‐enzymes, producing a range of small molecules, which include 2′‐(5″‐phosphoribosyl)‐5′‐ADP (pRib‐ADP) or pRib‐AMP, as well as ADP‐ribosylated ATP/ADPR (ADPr‐ATP/diADPR; Huang *et al*, 2022; Jia *et al*, 2022; Fig 3). These small molecules are, in turn, perceived by a downstream signaling hub, comprised of the lipase‐like proteins EDS1 (enhanced disease susceptibility 1), PAD4 (phytoalexin deficient 4), and SAG101 (senescence‐associated gene 101). Upon activation of upstream TIR‐NLRs, EDS1 forms mutually exclusive heterodimers with SAG101 or PAD4. The small molecule profile generated by the upstream activated TIR‐NLRs determines which EDS1 heterodimer is formed (Huang *et al*, 2022; Jia *et al*, 2022; Fig 3). The EDS1 signaling hub activates downstream helper CC_R_‐NLRs, NRG1, and ADR1, with these helpers ultimately mediating the induction of cell death. EDS1‐SAG101 heterodimers form a complex with NRG1, leading to its oligomerization into an NRG1‐EDS1‐SAG101 complex (Sun *et al*, 2021; Feehan *et al*, 2023; Fig 3). Although multiple groups have independently reported oligomerization of activated NRG1 (Jacob *et al*, 2021; Feehan *et al*, 2023; preprint: Wang *et al*, 2023b), whether EDS1‐SAG101 associates with NRG1 oligomers stably, transiently, or in a timepoint‐dependent manner, remains to be determined.

In contrast to EDS1‐SAG101, EDS1‐PAD4 heterodimers associate with ADR1 and ADR1‐like proteins (Huang *et al*, 2022). Activated NRG1 and ADR1 complexes then associate with the plasma membrane to act as calcium channels (Jacob *et al*, 2021; Saile *et al*, 2021; preprint: Wang *et al*, 2023b). Interestingly, NRG1 and ADR1 exhibit functional specialization. Not all TIR‐NLRs can signal through both helpers (Castel *et al*, 2019; Wu *et al*, 2019; Fig 3). Moreover, NRG1 and ADR1 contribute differentially to immunity. While both mediate transcriptional reprogramming downstream of activation, NRG1 appears to be partially specialized in cell death induction, while ADR1 contributes to basal immunity and defense, independently of cell death (Saile *et al*, 2020). Considering that different TIR‐NLRs activate different downstream helper CC_R_‐NLRs, how exactly the enzymatic activity of different TIR‐NLRs is decoded by the EDS1 node is not yet clear. Moreover, the functional determinants of diversification and the interplay between NRG1 and ADR1 in immunity is not fully understood.

### Cell biology of NLR resistosomes

Although we still lack a detailed understanding of the cell biology of these immune receptors, much progress has been made in recent years. NLRs can exhibit distinct subcellular localizations in their inactive as well as their activated states, as reviewed in depth by Lüdke *et al* (2022) and Shepherd *et al* (2023). For sensor NLRs, these localizations can be rationalized with the need to efficiently detect effector molecules, which in turn can target distinct subcellular compartments in the host (Wang *et al*, 2018; Duggan *et al*, 2021; Petre *et al*, 2021). One well described example is the previously discussed TIR‐NLR pair RRS1/RPS4, which recognizes the activity of effectors that manipulate WRKY TFs (Deslandes *et al*, 2002; Le Roux *et al*, 2015; Sarris *et al*, 2015). The inhibited RRS1/RPS4 complex associates with chromatin in the nucleus and is activated upon effector interaction and modification of the RRS1 WRKY domain (Birker *et al*, 2009; Narusaka *et al*, 2009; Williams *et al*, 2014; Le Roux *et al*, 2015; Sarris *et al*, 2015; Huh *et al*, 2017). Moreover, some sensor NLRs are required to associate with host guardees or decoys, which themselves may exhibit specific subcellular localizations. Examples include the well‐known CC‐NLRs RPM1 and RPS5, both of which guard plasma membrane‐associated host immune components and therefore require plasma membrane localization prior to activation to be functional (El Kasmi *et al*, 2017; Pottinger & Innes, 2020).

In the NRG1/ADR1 network, helper NLRs also exhibit diverse localizations and have been shown to dynamically relocalize upon activation. The ADR1 and NRG1 families of helper CC_R_‐NLRs, which act downstream of TIR‐NLRs, were both shown to reside in the cytoplasm as well as in the PM in their inactive states (Saile *et al*, 2021). In addition, inactive NRG1A and NRG1B were also reported to localize to the endoplasmic reticulum (Wu *et al*, 2019). Upon TIR‐NLR signaling, ADR1 and NRG1 both form higher molecular complexes that shift their localization, forming puncta at the plasma membrane (Saile *et al*, 2021; Feehan *et al*, 2023). Both the localization and functionality of NRG1 and ADR1 helpers are phospholipid dependent, as depletion of phosphatidylinositol‐4‐phosphate results in mislocalization and a loss of cell death activity (Saile *et al*, 2021; preprint: Wang *et al*, 2023b). However, a relocalization of NRG1 to the nucleus was also reported upon activation (Feehan *et al*, 2023). It remains to be determined what the direct function of NRG1 in the nucleus could be. However, the shift from a cytoplasmic to a membrane‐associated localization has also been described for the singleton NLR ZAR1 (Bi *et al*, 2021), further outlining that the subcellular shift toward the plasma membrane could be a general feature of singleton and helper NLRs. Whether this is related to the calcium channel activity of the resistosomes remains unclear (see also Box 1).

In the NRC network, the localization of sensors and helpers also plays an important role. The R protein Rpi‐blb2 is an NRC4‐dependent sensor NLR that confers resistance to strains of the oomycete *Phytophthora infestans* carrying the effector AVRblb2 (Bozkurt *et al*, 2011; Wu *et al*, 2017). Recently, Duggan *et al* (2021) showed that during infection with *P*. *infestans* and in the absence of activation, NRC4 focally accumulates at the extra‐haustorial membrane (EHM), where effectors are delivered into the host cell, and AVRblb2 accumulates during pathogen infection (Duggan *et al*, 2021). Following activation, NRC4 loses this focal perihaustorial localization and accumulates as puncta spread throughout the PM, triggering cell death presumably due to the formation of oligomeric resistosomes (Duggan *et al*, 2021; Contreras *et al*, 2023a,b). Distinct NLRs display a degree of specificity in their localization given that focal haustorial accumulation is specific to NRC4 and not its paralog NRC2 nor the singleton CC‐NLR ZAR1 (Duggan *et al*, 2021). Sensors co‐evolve with their matching effectors and may have to adapt to their localization to capture the effector. Helpers, in turn, may also evolve specialized localization patterns to match their sensors and enable effective sensor signaling. Interestingly, most *P. infestans* sensors characterized to date are NRC4‐dependent, so it is perhaps not surprising that NRC4 has acquired a focal localization around an infection structure produced by this oomycete pathogen (Wu *et al*, 2018; Derevnina *et al*, 2021). In addition, the NRC2 helper forms fibril‐like structures at the EHM during the interaction with *P. infestans* and may therefore contribute in a different way to immunity against *P. infestans* (Duggan *et al*, 2021).

Although the details of how networked CC‐NLR sensors communicate and activate their downstream helpers remain unclear, it can be assumed that a certain degree of subcellular proximity is required for efficient signal transmission. Recent work on the sensor Rx and its helper NRC2 demonstrates that they co‐localize in the cytoplasm in the absence of the coat protein effector molecule from *Potato virus x* (Contreras *et al*, 2023b). Activation of the system leads to a shift in localization to plasma membrane puncta for the NRC2 helper resistosome, while Rx remains cytoplasmic (Contreras *et al*, 2023b). The formation of puncta and a relocalization to the plasma membrane have also been reported for activated ZAR1 and NRC4 (Duggan *et al*, 2021).

What determines the subcellular localization of helper NLRs pre‐ or post‐activation? Domain swap experiments involving the N‐terminal CC‐domain of NRC2 and NRC4 demonstrated that this can lead to a shift in localization for these helper NLRs in their inactive state (Duggan *et al*, 2021). Thus, the N‐terminal CC domain appears to play an important role in determining the localization of NLR helpers, possibly due to interaction with membranes of various compositions. Moreover, it is still unclear what the nature or function of the observed punctate structures are or whether they represent molecular condensates or macro‐complexes of activated resistosomes. The diversity of NLR localizations and their dynamic relocalization following receptor activation is another example of the remarkable functional diversity exhibited by these proteins (see also Box 1).

### Atypical NLRs: modulators of plant immunity

An emerging concept in NLR network biology is that NLRs can modulate the activity of other NLRs in configurations that do not match the conventional model of sensor–helper pairing. The first reports of this phenomenon were classic examples of genetically linked sensor–helper pairs where the sensor can suppress a constitutively active helper, as is the case for the RGA5/RGA4 and RRS1/RPS4 pairs discussed above (Césari *et al*, 2014; Ma *et al*, 2018). More recently, two studies identified NLR modulators that cannot be classified into the conventional categories of sensor or helper. Rather, these atypical NLRs function as modulators of NLR signaling (Wu *et al*, 2022; Adachi *et al*, 2023a). The *NRG1* NLR gene cluster in *Arabidopsis* consists of *NRG1a*, *NRG1b*, and *NRG1c*, also termed *NRG1.1*, *NRG1.2*, *and NRG1.3* respectively (Wu *et al*, 2022). As described above, NRG1A and B proteins are well‐known helper NLRs required for immune signaling and the induction of cell death downstream of TIR‐NLRs. Both NRG1A and NRG1B contain all the features of canonical NLRs an N‐terminal CC_R_ domain, a central NB‐ARC, and a C‐terminal LRR domain. NRG1C, on the other hand, is a truncated NLR lacking a CC_R_ domain and most of the NB‐ARC, thus making it unlikely to function as a helper NLR. In contrast, NRG1C can negatively regulate NRG1A/B signaling and cell death, presumably by competing with NRG1A/B proteins for interaction with EDS1/SAG101 complexes. However, NRG1C can also negatively regulate autoimmune variants of NRG1A that trigger cell death in an EDS1‐independent manner, meaning that the precise mechanism by which NRG1C immunomodulates NRG1A and B requires further investigations (Wu *et al*, 2022; Fig 3).

Another NLR modulator is NRCx, a full‐length CC‐NLR that phylogenetically clusters with helper NRCs and is closely related to NRC2 and NRC3. Similar to other NRCs, NRCX has the classic tripartite domain architecture of CC, NB‐ARC, and LRR domains. However, unlike other NRCs, the NRCx N‐terminal MADA sequence is nonfunctional and unable to trigger cell death when swapped for functional MADA N‐termini (Adachi *et al*, 2023a). NRCx is also unusual for an NLR, as its silencing results in autoimmunity in *N. benthamiana*. This autoimmunity is partially dependent on the NRC family members NRC2 and NRC3, but not NRC4, with NRCX silencing leading to enhanced cell death by NRC2 and NRC3, but not NRC4 (Adachi *et al*, 2023a). This points to NRCX as an immunomodulating component in the NRC network, specifically capable of regulating the functions of NRC2 and NRC3 (Fig 3). Considering its evolutionary relatedness with NRC2 and NRC3, it is tempting to speculate that NRCX arose during NRC evolution to temper the activity of these NRCs, possibly during particular stages of plant development, and to avoid inappropriate activation of NRC2/3‐dependent pathways (Kourelis *et al*, 2022; Adachi *et al*, 2023a). The precise biochemical mechanisms by which NRCX negatively regulates the NRC2 and NRC3 helpers remain unknown and await further research.

NRG1C and NRCx are atypical NLRs because they act as NLR modulators within their respective NLR networks. It is possible that the complex network configurations that arose throughout evolution resulted in novel NLR functional specializations to maintain network homeostasis and block misregulation. Genetically dispersed NLR networks are probably more prone to mis‐activation and may be more difficult to regulate; therefore, NLR modulators may have emerged to provide these increasingly complex signaling architectures with additional regulation (see also Box 1). Understanding the molecular mechanisms by which modulator NLRs function will shed light on NLR network regulation. Moreover, this knowledge may enable new avenues for bioengineering more efficient signaling in plant immunity by altering the function, intensity, or specificity of the immunomodulators.

### Tissue and cell specificity of NLR proteins

Effective deployment of plant immunity can be achieved through tissue‐ and cell‐specific responses. However, in sharp contrast to animal immunity (Weisberg *et al*, 2021), our understanding of plant immunity, including NLR biology, at the tissue and cell level is limited. Only a few studies examined the expression patterns of NLR genes across different plant tissues. In *Arabidopsis*, the majority of NLRs are more highly expressed in the shoot compared to the root tissue. In contrast, legumes and monocots have overall higher expression levels of NLR genes in roots compared to shoots (Munch *et al*, 2018). Tissue‐ and organ‐specific NLR expression patterns may reflect a resistance response to pathogens that attack specific tissues (Bhardwaj *et al*, 2011; Wang *et al*, 2011). Tissue‐specific expression of NLR genes may also reduce the risk of inadvertent activation of immune responses in certain tissue types, given that NLR mis‐regulation can lead to adverse effects (Bomblies *et al*, 2007; Palma *et al*, 2010).

Tissue‐specific upregulation of the expression of immune receptors could contribute to an effective and specific response to pathogen attack. NLRs, especially TIR‐type NLRs, can show induced expression during pathogen infection, thereby enhancing general immunity or priming the immune system (Mohr *et al*, 2010; Yu *et al*, 2013; Tian *et al*, 2021). Similarly, Adachi *et al* (2023a) reported that some NRC helper genes are induced following pathogen infection. NRC2a and NRC2b were upregulated following treatment of *N. benthamiana* leaves with the nonpathogenic *Pseudomonas fluorescens*, while other NRC genes such as NRC3 and NRC4a/b and the immunomodulator NRCx remained unchanged. This marked shift in balance of NRC2a/b to NRCx expression may contribute to more robust immune responses (Adachi *et al*, 2023a). RNA‐sequencing of *N. benthamiana* roots, leaves, and flowers/buds revealed that while NRC2, NRC3, NRC4, and NRCx were expressed in all three tissues, other NRCs, such as NRC7a and NRC8b, were mainly expressed in roots and not in other tissues. (Adachi *et al*, 2023a). How potential tissue‐ or cell‐type‐specific expression and induction of NLR genes contribute to enhancing the robustness and efficiency of plant immune responses is not yet understood.

To what degree do NLRs exhibit cell‐type specificity (see also Box 1)? With the advent of single‐cell RNA‐sequencing techniques, cell‐specific expression patterns of immune receptors in plants can be elucidated. A recent preprint by Tang and colleagues (preprint: Tang *et al*, 2023) demonstrated that in *Arabidopsis*, some TIR‐NLR proteins show specific expression in cells of the vasculature. In addition, treatment with the fungal pathogen *Colletotrichum higginsianum* resulted in upregulation of NLRs specifically in vascular tissues (preprint: Tang *et al*, 2023). These observations would be in line with cell‐layer‐specific immune responses observed in plant roots under pathogen threat (Chuberre *et al*, 2018; Rich‐Griffin *et al*, 2020; Fröschel *et al*, 2021) and the vascular‐specific hypersensitive cell death observed in Brassicaceae against the bacterium *Xanthomonas campestris* (Kamoun *et al*, 1992).

The mechanisms that drive tissue‐ or cell‐type‐specific expression patterns remain largely unknown. In addition to specific promoters and epigenetic regulation of expression (Tsuchiya & Eulgem, 2013; Le *et al*, 2015), NLR transcript levels are also influenced by small RNAs (Zhai *et al*, 2011; Zhang *et al*, 2016). Moreover, specific E3 ligases can also control protein levels of NLRs post‐transcriptionally (Wu *et al*, 2020). Whether these mechanisms can create tissue‐ or cell‐type‐specific patterns of NLRs remains to be tested. The elucidation of NLR expression patterns at the cellular resolution will further our understanding of NLR networks and enable a more efficient deployment of NLRs in agriculture.

### Crosstalk between cell surface immune receptors and NLRs

In addition to NLRs, plants detect pathogens through cell surface pattern recognition receptors (PRRs), which consist of two major classes of transmembrane proteins: receptor proteins (RPs) and receptor kinases (RKs). RPs carry a small cytoplasmic tail, but unlike RKs, they lack a C‐terminal kinase domain. Similar to NLRs, RPs and RKs are also known to form receptor networks (Smakowska‐Luzan *et al*, 2018; Wu *et al*, 2018). Following perception of pathogen‐derived molecules, RPs and RKs hetero‐oligomerize with co‐receptor RKs, such as the LRR‐RK SERK3/BAK1, which activates downstream signaling by phosphorylating downstream RLCKs to result in an immune response, generally known as PRR‐triggered immunity (PTI; Ngou *et al*, 2022a; Zhang *et al*, 2023).

Traditionally, PRRs and NLRs were thought to induce distinct immune pathways. However, there is intricate crosstalk between the signaling pathways induced by cell‐surface and intracellular immune receptors. Indeed, PRRs and NLRs mutually potentiate each other by enhancing transcription of the core signaling components of these pathways (Ngou *et al*, 2021; Yuan *et al*, 2021). Specifically, the capacity of some NLRs to activate hypersensitive cell death in response to effectors is compromised in the absence of cell surface immune signaling. Examples include activation of the RRS1/RPS4 TIR‐NLR pair by the bacterial effector AvrRps4, activation of the CC_G10_‐NLRs RPS2 and RPS5 by AvrRpt2 and AvrPphB, respectively, and activation of the CC‐NLR RPM1 by AvrRpm1 (Ngou *et al*, 2021; Yuan *et al*, 2021). Recent work has shown that cell‐surface and intracellular immune receptor repertoires across plant species concertedly expand and contract. This suggests that they are potentially co‐evolving and/or functionally interdependent, in support of the mutual potentiation model mentioned above (Ngou *et al*, 2022b).

NLRs can also act genetically downstream of cell surface immune receptor activation. In *Arabidopsis*, the EDS1/PAD4/ADR1 and, to a lesser extent, the EDS1/SAG101/NRG1 modules are genetically required for a subset of the immune responses triggered by LRR‐RPs and LRR‐RKs (Pruitt *et al*, 2021; Tian *et al*, 2021; Fig 3). Some cell‐surface RPs can trigger a hypersensitive response that is indistinguishable from the NLR‐mediated cell death response. In tomato, the RPs Cf‐4, Cf‐9, Cf‐2, and Cf‐5 recognize apoplastic secreted effectors of the fungus *Cladosporium fulvum*, while Ve1 recognizes the effector Ave1 from fungi of the *Verticillium* genus (Luderer *et al*, 2002; Seear & Dixon, 2003; Rivas & Thomas, 2005; De Jonge *et al*, 2012). Helper NLRs of the NRC family are required for the hypersensitive cell death mediated by these RPs (Gabriëls *et al*, 2006, 2007; Fradin *et al*, 2009; Kourelis *et al*, 2022). Recent work by Kourelis *et al* (2022) involving a combination of *nrc2/3/4* KO plants and genetic complementation revealed that NRC3 is the primary helper NLR required for the hypersensitive response induced by the Cf receptors (Fig 3). EDS1 was also shown to be required for full Ve1 and Cf4‐mediated immunity, implying that the NRG1/ADR1 network may also be involved in signaling for these cell‐surface receptors (Fradin *et al*, 2009; Kourelis *et al*, 2022). If this is the case, at least two distinct NLR networks have evolved to contribute to cell‐surface signaling in solanaceous plants. Although it is tempting to speculate that sensor NLRs act as a bridge between cell‐surface receptors and downstream helpers, the precise molecular link between cell‐surface immunity and downstream helper NLRs for Cfs and Ve1 is not known (see also Box 1).

Considering the importance of cell‐surface immunity and its synergy with intracellular immune responses, it is not surprising that NLRs have evolved to guard cell‐surface receptors and their downstream components. As mentioned previously, the CC‐type NLRs ZAR1 and Prf have evolved to recognize bacterial effectors by interacting with RLCKs. ZAR1 engages with subfamily XII‐2 RLCKs, also called ZRKs, in *Arabidopsis* and *N. benthamiana*, while Prf monitors the Pto kinase in tomatoes (Salmeron *et al*, 1996; Bombarely *et al*, 2012; Lewis *et al*, 2013; Ntoukakis *et al*, 2014; Wang *et al*, 2015; Seto *et al*, 2017; Schultink *et al*, 2019; Laflamme *et al*, 2020; Adachi *et al*, 2023b). The *Arabidopsis* NLR RPS5 and the unrelated barley (*Hordeum vulgare*) NLR Pbr1 have converged on guarding PBS1‐like RLCKs to recognize the bacterial effector AvrPphB (Carter *et al*, 2019). Recently, the CSA1/CHS3 TIR‐NLR pair was shown to guard BAK1 and BIR3 homeostasis, triggering cell death upon sensing its perturbation (Schulze *et al*, 2022). Interestingly, cell death induced by the microbial pattern pg23 was found to be CSA1‐dependent (Schulze *et al*, 2022; Yang *et al*, 2022; Fig 3). It is possible that NLR‐dependence for cell death or immunity of some PRRs could arise from guard‐guardee NLR dynamics, where singleton or sensor NLRs evolve to guard components of cell‐surface immunity and subsequently become necessary for the full function of their PRR guardees.

While much progress has been made in recent years toward understanding the functional links between cell‐surface and intracellular immunity, further studies should strive to study PRRs and NLR receptors with an appreciation of their huge structural and functional diversity. It is evident that distinct classes of cell‐surface and intracellular receptors interact with each other to different degrees and through different mechanisms. Moreover, it appears that many of the connections between cell‐surface and intracellular receptors may vary across species, while others are conserved (Kourelis *et al*, 2022). The full picture of the complex and interdependent nature of plant cell‐surface and intracellular immune receptor networks will only be clarified once we integrate their structural and evolutionary diversity.

### Pathogen suppression of NLRs by modulation of immune signaling pathways

**Figure 4:**
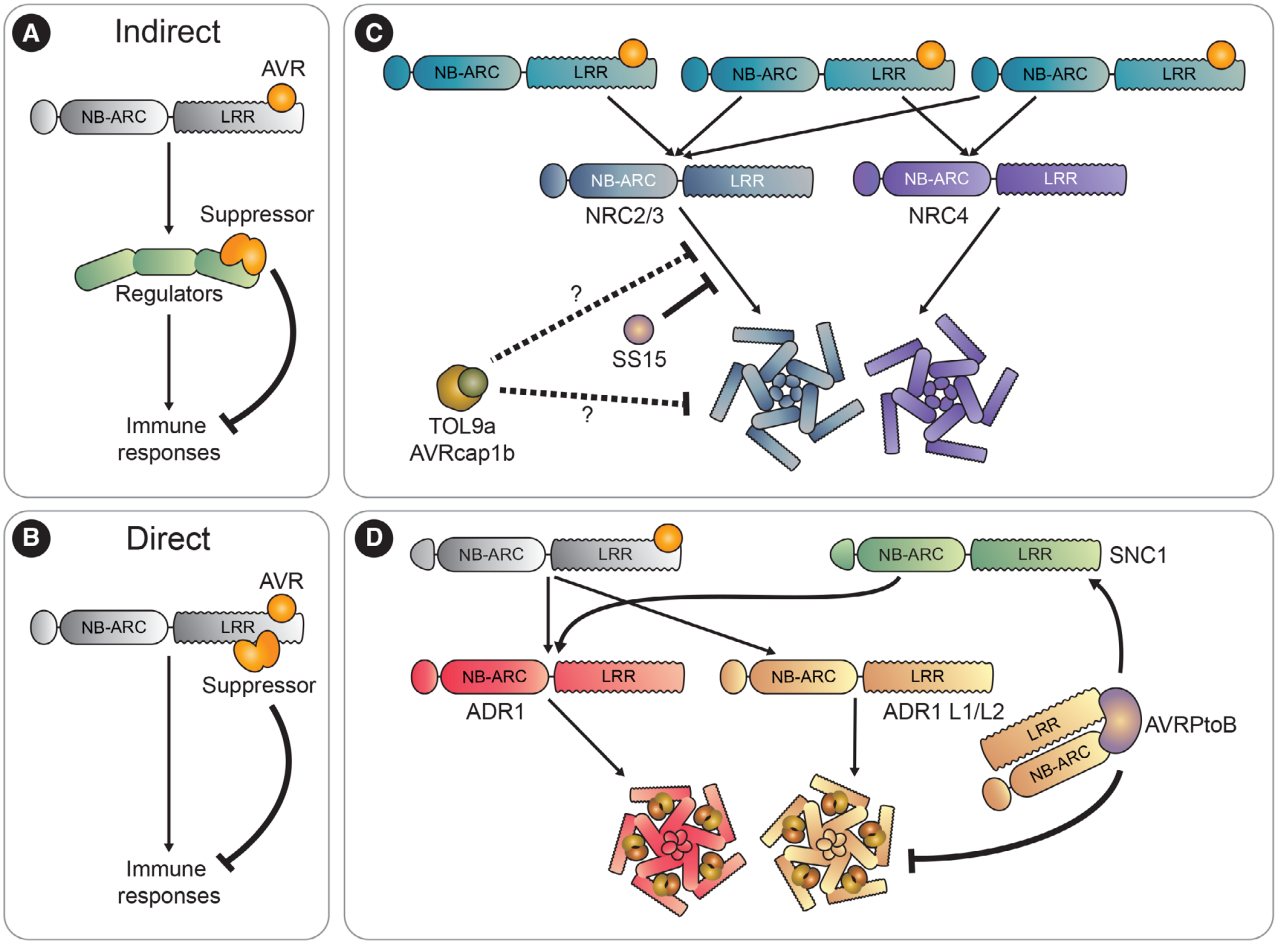
Mechanisms of pathogen suppression of NLR immune signaling Pathogen effectors can suppress NLR signaling via diverse strategies. Some effectors can target downstream components required for NLR signaling to suppress immune signaling (Table 1) (A), while others can suppress immune responses by directly targeting NLRs (B). (C) In the NRC network, two effectors have been identified that can suppress immune signaling of multiple sensor NLRs by directly targeting their downstream helpers, NRC2 and NRC3 (Derevnina *et al*, 2021). The potato cyst nematode effector SS15 acts as an inhibitor of NRC2/3, directly binding their NB‐ARC domain to prevent their oligomerization (Contreras *et al*, 2023b). In contrast, the potato late blight pathogen effector AVRcap1b interacts with the host protein NbTOL9a, a vesicle trafficking component, to suppress NRC2/3‐mediated cell death via an unknown mechanism. NRC4 is not suppressed by these two effectors and can mediate immune signaling by many upstream sensors even in the presence of SS15 and AVRcap1b, highlighting the robustness provided by helper NLR redundancy in these networked architectures. The question mark (?) indicates that the exact mechanism by which these proteins (AVRcap1b and TOL9a) modulate the other proteins in the figure (NRC2/3, specifically), is not known. (D) The bacterial effector AvrPtoB can suppress ADR1‐L1 and ADR1‐L2 by acting as an E3 ligase, targeting these helper NLRs for degradation (preprint: Wang *et al*, 2023a). AVRPtoB cannot suppress ADR1, another helper NLR. The sensor NLR SNC1 guards ADR1‐L1/L2 and, upon detecting the activity of AvrPtoB, can activate immunity via ADR1, another example of how helper NLR network configuration can confer robustness to pathogen suppression.

An emerging concept in NLR biology is that pathogen effectors can act as both triggers and suppressors of NLR‐triggered immunity. While most effectors studied to date suppress immune pathways induced by PTI, in some cases, adapted pathogens deploy effectors to interfere with host NLR signaling to promote disease (Wu & Derevnina, 2023). This effector‐mediated suppression of NLR signaling can be achieved by various mechanisms. Some effectors act indirectly by interfering with host proteins that contribute to NLR signaling pathways, while others act directly by physically interacting with NLRs to perturb their function (Lopez *et al*, 2019; Derevnina *et al*, 2021; Karki *et al*, 2021; preprint: Wang *et al*, 2023a; Fig 4A and B).

The effector AVRcap1b from *P. infestans* can indirectly suppress cell death mediated by the *N. benthamiana* helper NLRs NRC2 and NRC3 by physically interacting with the host protein NbTOL9a, a component of the endosomal sorting complex required for transport (ESCRT) vesicle trafficking machinery (Moulinier‐Anzola *et al*, 2020; Derevnina *et al*, 2021). NbTOL9a was shown to act as a negative regulator of NRC2/3‐mediated cell death (Derevnina *et al*, 2021). While the exact mechanism by which AVRcap1b‐NbTOL9a interaction leads to suppression of NRC function is unknown, AVRcap1b is presumably hijacking a negative regulator to compromise NRC2/3‐mediated immunity activities (Fig 4C).

Another example is the *Xanthomonas euvesicatoria* effector XopQ, which can suppress NLR signaling by targeting and directly binding TFT4, a protein of the 14–3‐3 family that functions in immunity downstream of NLR activation, presumably by interfering with TFT4‐client interactions required for correct immune signaling (Teper *et al*, 2014).

The effector RipAC from the bacteria *R. solanacearum* interferes with NLR signaling by associating with SGT1, an important host regulator required for NLR homeostasis and function. By forming a complex with SGT1, RipAC prevents MAPK‐mediated phosphorylation of SGT1, which is normally enhanced upon immune activation (Yu *et al*, 2020). RipAC‐SGT1 complex formation also prevents association between SGT1 and RAR1. SGT1, RAR1, and HSP90 normally form a molecular chaperone ternary complex, which is required for the correct functionality of multiple NLRs (Azevedo *et al*, 2006). This perturbation of SGT1 phosphorylation and SGT1‐RAR1 complex formation by RipAC can suppress immunity mediated by two SGT1‐dependent NLRs, R3a, and RPS2, as well as immune signaling triggered by the *R. solanacearum* AVR effectors RipAA and RipP1 (Nakano *et al*, 2020; Yu *et al*, 2020).

Another remarkable example is the HopBF1 effector from *P. syringae*. This effector can mimic a host client of the NLR chaperone HSP90, binding HSP90 and phosphorylating it, which results in its complete inactivation. As this chaperone is required to maintain NLRs in a stable, inactive, and signal‐competent form, this phosphorylation results in compromised NLR signaling (Lopez *et al*, 2019).

Additional effectors that inhibit or suppress NLR pathways have been identified to date and are listed in Table 1. However, in most cases, their precise molecular mechanisms and host targets are not yet fully known.

**Table 1:** Summary of known NLR‐suppressing effectors, their host targets, and activities.

Effector	Pathogen	Supressed NLRs	Host target	Effector activity	References
HopZ3	*P. syringae*	RPM1	RPM1	Acetylation of host RPM1 results in inactivation of immune complex	Lee *et al* (2015)
HopBF1	*P. syringae*	RPM1	HSP90	Phosphorylation of host HSP90 results in its inactivation	Lopez *et al* (2019)
AvrPtoB	*P. syringae*	ADR1‐L1, ADR1‐L2	ADR1‐L1, ADR1‐L2	Ubiquitinates and promotes degradation of helper CC_R_‐NLRs, ADR1‐L1, and ADR1‐L2	preprint: Wang *et al* (2023a)
XopQ	*X. euvesicatoria*	Pto, Gpa2	TFT4	Binding to host TFT4 prevents association with downstream immune‐related targets	Saunders *et al* (2012)
RipAC	*R. solanacearum*	R3a, RPS2	SGT1	Association prevents phosphorylation by MAP kinases	preprint: Yu *et al* (2019)
Lso‐HPE1	*C. liberibacter*	Prf	Unknown	Unknown	Levy *et al* (2019)
RHA1b	*G. pallida*	Prf, Rx, Rpi‐blb1, Gpa2, Bs4	Unknown host E2 ubiquitin conjugation enzymes	Ubiquitination of target NLRs prevents their accumulation	Kud *et al* (2019)
SS4, SS18, SS19	*G. rostochiensis*	Rx	Unknown	Unknown	Ali *et al* (2015)
SS15	*G. rostochiensis*	All NRC2/3‐dependent sensor NLRs	NRC2, NRC3	Binds NB‐ARC domain to prevent activation and oligomerization	Ali *et al* (2015); Contreras *et al* (2023a); Derevnina *et al* (2021)
SPRYSEC34, SS10	*G. rostochiensis*	Rpi‐blb2	Unknown	Unknown	Ali *et al* (2015); Derevnina *et al* (2021)
IPI‐O4	*P. infestans*	RB (also known as Rpi‐blb1)	RB	Binding to CC‐domain of RB immune signaling	Chen *et al* (2012); preprint: Karki *et al* (2020)
PITG‐15278	*P. infestans*	Rpi‐blb2	Unknown	Unknown	Derevnina *et al* (2021); Oh *et al* (2023)
Avr1	*F. oxysporum f. sp. lycopersici*	I2 and I3	Unknown	Unknown	Houterman *et al* (2008)

### Pathogen suppression of NLRs by direct inhibition

Some effectors can suppress immunity by directly targeting NLR proteins (Fig 4B, Table 1). The effector HopZ3 from *P. syringae* is a YopJ family acetyltransferase that acetylates members of the CC‐NLR RPM1 immune complex, thereby inactivating its immune response and promoting pathogen growth (Lee *et al*, 2015). Another example is the RHA1B effector from the root knot nematode *Globodera pallida*, which functions as an E3 ubiquitin ligase that targets NLRs for degradation, thereby suppressing immunity (Kud *et al*, 2019). More recently, additional studies have revealed that pathogen effectors can directly target helper NLRs to suppress immunity.

Two studies on the SS15 effector from the potato cyst nematode *Globodera rostochiensis* (Derevnina *et al*, 2021; Contreras *et al*, 2023a) revealed that SS15 can inhibit cell death triggered by the helper NLRs NRC2 and NRC3, as well as the tomato (*Solanum lycopersicum*) helper NLR NRC1 of the solanaceous NRC network (Fig 4C). SS15 acts as a proteinaceous inhibitor, directly binding to the HD1 region of the central NB‐ARC domains of the helper NLRs (Derevnina *et al*, 2021; Contreras *et al*, 2023a). SS15 was proposed to immobilize a critical “hinge” loop in the NB‐ARC domain, preventing intramolecular rearrangements required for activation and resistosome formation. As a consequence, the inhibitor locks NRC proteins in an inactive conformation and prevents the oligomerization and plasma membrane translocation that otherwise would follow activation by upstream NRC‐dependent sensors (Contreras *et al*, 2023a).

A recent study on the bacterial effector AvrPtoB revealed that it can target helper NLRs in the NRG1/ADR1 network for degradation. AvrPtoB has E3 ligase activity and can ubiquitinate and promote degradation of ADR1‐L1, while having similar but milder effects on ADR1‐L2 (preprint: Wang *et al*, 2023a). This activity suppresses ADR1‐L1 and ADR1‐L2‐mediated cell death (Fig 4D). Interestingly, AvrPtoB cannot suppress ADR1, and this specificity in helper NLR suppression appears to be encoded in the N‐terminal CC_R_ domain (preprint: Wang *et al*, 2023a).

These two studies indicate that targeting helper NLRs that are central nodes in NLR networks is a common virulence strategy (Contreras *et al*, 2023a; preprint: Wang *et al*, 2023a; Fig 4C and D). The fact that three distantly related pathogens, an oomycete, a nematode and a bacterium, have convergently evolved effectors that suppress networked helper NLRs highlights how important NLR networks are in mediating immunity against diverse pathogens (Derevnina *et al*, 2021; preprint: Wang *et al*, 2023a). Targeting helper NLRs is a favorable virulence mechanism for pathogens, given that shutting down one helper node results in simultaneously compromising multiple upstream sensor NLRs and cell surface receptors. This was demonstrated for AVRcap1b and SS15, since both could suppress cell death triggered by the tomato cell‐surface RP Cf‐4 upon recognition of the fungal effector Avr4, in addition to suppression of sensor NLRs (Kourelis *et al*, 2022).

Indeed, networked signaling architectures are thought to increase the robustness of the plant immune system (Adachi & Kamoun, 2022), and redundant nodes in the form of helper NLRs may help to evade suppression by pathogen effectors. Notably, in all three cases, the host plant features additional “backup” helper nodes that are insensitive to the effectors. For instance, both SS15 and AVRcap1b can suppress *N*. benthamiana NRC2 and NRC3, but not their NRC4 paralog, whereas AvrPtoB can suppress ADR1‐L1 and ADR1‐L2, but not ADR1 in *Arabidopsis* (Derevnina *et al*, 2021; preprint: Wang *et al*, 2023a).

It is possible that some pathogens have evolved effectors that suppress all helper nodes simultaneously, therefore compromising an entire network in a given plant taxon. Such pathogens would then have a wide host range, and this could explain the anomalously extended host range of pathogens like *Phytophthora palmivora*, which has > 100 host plant species (Erwin & Ribeiro, 1996; Carella *et al*, 2018). However, it should be noted that SS15, AVRcap1b, and AvrPtoB also have AVR activities, meaning that they are recognized by particular plant genotypes. SS15 triggers cell death mediated by an unknown NLR in *Nicotiana tabacum* (Ali *et al*, 2015). AVRcap1b is recognized by Rpi‐cap1 in *Solanum capsicibaccatum*, and while the underlying gene is unknown, it is hypothesized to be a TIR‐type NLR (Verzaux *et al*, 2012). That would imply that Rpi‐cap1 would recognize AVRcap1b through a signaling pathway that is independent of the NRC CC‐NLR network (Rietman, 2011; Verzaux *et al*, 2012). This would be analogous to the TIR‐NLR SNC1, which recognizes the effector AvrPtoB. By guarding ADR1‐L1/L2 helpers targeted for suppression by AvrPtoB, SNC1 activates immune signaling via ADR1, a helper that is insensitive to AvrPtoB (preprint: Wang *et al*, 2023a; Fig 4D). Therefore, NLR suppressing effectors can be recognized and in addition, just like other important immune nodes, helper NLRs can be guarded by particular NLRs in order to prevent pathogen interference.

### Effector suppression of NLRs: the dark matter of R gene discovery?

How many R genes are present cryptically in crop genomes due to pathogen suppression? This is an exciting unanswered question raised by the discovery of effectors that suppress helper NLRs (see also Box 1). R‐gene discovery may have overlooked potential sources of resistance due to the presence of hitherto unknown NLR suppressors in the pathogen. This R‐gene masking becomes even more of an issue whenever pathogens suppress helper NLRs, which are downstream nodes in networks of sensor NLRs and cell surface receptors (Derevnina *et al*, 2021; Contreras *et al*, 2023a; preprint: Wang *et al*, 2023a).

Pathogen NLR suppressors may also explain cases in which NLR genes could not be successfully transferred between plant families, a phenomenon known as restricted taxonomic functionality (RTF; Tai *et al*, 1999). While in some cases RTF may be due to missing genetic components in the heterologous plant, it is possible that species‐specific pathogen suppressors could underpin the lack of functionality of some NLR across taxa. Future work should consider NLR‐suppressing effectors and their potential contributions to RTF in R‐gene discovery and disease resistance breeding pipelines in order to avoid overlooking a potentially vast and untapped reservoir of immune receptors.

### NLR bioengineering: new recognition specificities

The deployment of R‐genes in agriculture is often ineffective as pathogens tend to rapidly evolve to evade recognition. In addition, few R genes have been identified against the majority of economically important pathogens. This has fueled attempts to bioengineer made‐to‐order NLR immune receptors to achieve durable and versatile disease resistance (Farnham & Baulcombe, 2006; Segretin *et al*, 2014; Giannakopoulou *et al*, 2015; Kim *et al*, 2016; De la Concepcion *et al*, 2019; Huang *et al*, 2021; Liu *et al*, 2021; preprint: Wang *et al*, 2021; Cesari *et al*, 2022; Förderer *et al*, 2022; preprint: Maidment *et al*, 2022; Tamborski *et al*, 2023). This goal of designer R genes is often portrayed as the holy grail of plant pathology. Most attempts at NLR engineering to date have been aimed at obtaining novel disease resistance specificities, a topic that has been recently reviewed in depth by Marchal and colleagues (Marchal *et al*, 2022b). In particular, many approaches have focused on NLR‐ID engineering, specifically by resurfacing the structure of IDs by amino acid substitution or by swapping IDs for other closely related proteins to expand the effector recognition specificities of IDs (De la Concepcion *et al*, 2019; Liu *et al*, 2021; Bentham *et al*, 2023; Cesari *et al*, 2022; preprint: Maidment *et al*, 2022).

The ortholog/allelic series of the *Oryza* (rice) NLR pair Pia carries at least 9 different integrations (IDs) in the Pia‐2 (aka RGA5) sensor, indicating that throughout evolution, an NLR scaffold can accommodate the fusion of domains of unrelated sequence (Shimizu *et al*, 2022). Indeed, in the Poaceae, the majority of NLR‐IDs, including Pia‐2, belong to 24 major clades (major integration clades, or MIC; Bailey *et al*, 2018). This has inspired NLR bioengineers: If nature can make NLR‐IDs every few millions of years, can we not do this in our laboratories? (preprint: Schornack & Kamoun, 2023).

This view has even inspired bioengineers to integrate animal antibody domains into NLR proteins. In a recent proof‐of‐concept study, Kourelis and colleagues showed that the integrated HMA domain of the rice Pik‐1 sensor NLR can be replaced with camelid‐derived nanobodies, swapping the recognition specificity while retaining signaling via its downstream helper Pik‐2 (Kourelis *et al*, 2023). These engineered immune receptor pairs, termed pikobodies, can mount an effective immune response and virus resistance when activated with the fluorescent proteins (FPs) GFP and mCherry, leading to NLRs with completely synthetic recognition specificities. This work implies that NLRs could, in theory, be developed to recognize any antigen bound by nanobodies, combining animal adaptive immunity with plant innate immunity, and conferring a pseudo‐adaptive immune system to plants. Transgenic plants expressing pikobodies can confer resistance to FP‐expressing strains of *Potato virus X* (PVX) to levels comparable to the natural disease resistance gene Rx (a CC‐NLR; Kourelis *et al*, 2023). The pikobody bioengineering example highlights the importance of how fundamental knowledge of NLR function and evolution can guide approaches for disease resistance engineering. Indeed, the pikobody system is built on numerous studies on the genetics, biochemistry, and, particularly, the evolution of the Pik‐1/Pik‐2 NLR pair and its integrated domain (De la Concepcion *et al*, 2018, 2021; Zdrzałek *et al*, 2020; Białas *et al*, 2021). Nonetheless, we still do not understand the full mechanistic details of how the Pik‐1/Pik‐2 NLR pair becomes activated. Further advances on this topic will undoubtedly facilitate further bioengineering.

### NLR bioengineering: resistance gene resurrection

The occurrence of cryptic disease resistance genes in crop genomes as a consequence of effector suppression of NLRs opens up the possibility of engineering resilient immune receptors that evade pathogen inhibition. In a recent study, (Contreras *et al*, 2023a), the authors leveraged their mechanistic understanding of how SS15 interferes with NRC activation to develop “effector‐proof” NRC variants that evade SS15 suppression. To achieve this, they combined structural information from the inhibitor/NLR complex as well as sequence polymorphisms between inhibited vs. insensitive NLRs to generate a single amino acid variant of NRC2 that evades SS15 inhibition. This single amino acid variant has the capacity to activate cell death triggered by all tested upstream NRC2‐dependent sensors in the absence and presence of SS15 (Contreras *et al*, 2023a). This rescued upstream disease resistance gene is now “resurrected,” becoming able to respond to AVR effectors even when the inhibitor is present. This form of helper NLR bioengineering may be a highly effective strategy for achieving disease resistance, as it holds the potential of simultaneously restoring (resurrecting) functionality of multiple upstream sensors. In the future, it will be interesting to apply this bioengineering strategy to other NLRs that are suppressed by pathogen effectors. Moreover, because the engineering can involve single amino acid variants, this approach is amenable to gene editing, making it possible when transgenesis is not an option. It remains to be determined how durable resistance gene resurrection will be, considering that pathogens may evolve to counteract the introduced mutations.

## Discussion

In this review, we highlight some of the recent findings on NLR biology, covering how these immune receptors evolve, sense pathogens, and get activated. It is now becoming clear that our view of NLRs must move beyond single genetic and functional units to a receptor network perspective (Wu *et al*, 2018). The study of NLRs also needs to move beyond a few model plant species. Based on their RefPlantNLR database, Kourelis *et al* reported that, although approximately 500 NLRs have been experimentally validated, about 80% of seed plant clades still do not have a single NLR that has been experimentally confirmed. Future studies on NLRs will require a broader, phylogenetically informed view.

A hallmark of NLR biology and evolution is sub‐functionalization. While some singleton NLRs are individual functional units, the inter‐NLR cooperation and modulation exhibited in NLR pairs and in PRR‐NLR networks such as the NRC and NRG1/ADR1 network reveals that over evolutionary time, immune receptor specialization has likely led to new functions or degeneration for different NLR domains. The sensor–helper specialization of networked NLRs increases their versatility and evolvability, helping the plant immune system keep up with rapidly evolving pathogens. Moreover, the redundancy exhibited by these signaling networks makes them more robust and difficult to suppress by pathogens. As we uncover this genetic and functional NLR diversity, it is imperative to move beyond the simplified view of the NLR tripartite domain architecture and integrate the complexity of these higher order NLR signaling configurations into our understanding of NLR domain function and evolution to inform future research.

Ever since the cloning of the first NLR genes in the 1990s, the field has been hampered by the difficulty of studying activated NLR *in planta*, given that they trigger a cell death response. For CC‐NLRs, this has been addressed by the discovery of the N‐terminal MADA a1 helix, given that mutations in this motif abolish cell death activity without compromising immune receptor activation, plasma membrane association, or oligomerization. Many of the advances described in this article would not have been possible without these N‐terminal mutants due to the early onset cell death triggered by immune receptor activation. Nonetheless, even though these MADA‐motif mutants are a valuable tool to study activated CC‐NLRs, future studies will need to consider experiments with wild‐type sequences whenever possible.

While the new conceptual framework provided by the elucidation of the structure and activation mechanisms of various NLR resistosomes represents a major advance for the plant NLR field, there is still much work to be done in understanding how paired and networked NLRs function, with many important questions remaining unanswered (see also Box 1). What is the molecular basis of the evolutionary transition of NLRs from singletons to pairs and networks? How do sensors and helpers communicate and activate each other in NLR pairs and networks? How are immune receptor networks efficiently regulated? The interplay between cell‐surface receptors, sensor NLRs, helper NLRs, modulator NLRs, and the effectors that activate or suppress them is incredibly complex. Multi‐disciplinary approaches are required to disentangle how these PRR and NLR immune receptor networks function and evolve. Answering these questions holds the potential to deepen our understanding of the plant immune system and unlock a new era of disease resistance breeding (see also Box 1).

## Materials and Methods

### NLR identification and phylogenetic analyses for Fig 2

NLRtracker (Kourelis *et al*, 2021) was used to extract the NLRs from the proteomes of the *Arabidopsis thaliana* (GCF_000001735.4), *Glycine max* (GCF_000004515.6), *Solanum lycopersicum* (GCF_000188115.4), *Nicotiana benthamiana* (https://nbenthamiana.jp/), *Lactuca sativa* (GCF_002870075.2), *Oryza sativa* (GCF_001433935.1), *Hordeum vulgare* (GCF_904849725.1), *Spinacia oleracea* (GCF_002007265.1), and *Beta vulgaris* (GCF_002917755.1), representing four major plant phyla. Only proteins with canonical NLR domain architecture and an intact NB‐ARC domain were kept from NLRtracker output (“CNL”, “NL”, “CNLO”, “CONL”, “RNL”, “BCNL”, “BCNLO”, “TNL”, “TNLO”) and were deduplicated using CD‐HIT (−c 1.0; Fu *et al*, 2012). The NB‐ARC domains for the remaining sequences were extracted from NLRtracker output and aligned with RefPlantNLR (Kourelis *et al*, 2021) using MAFFT v7.490 (Katoh & Standley, 2013). The alignment was then used to construct an approximately maximum‐likelihood phylogenetic tree using FastTree v2.1.11 (default options; Price *et al*, 2009). The tree was visualized using iTOL (Letunic & Bork, 2021) and annotated manually. The species tree was obtained from the NCBI common taxonomy tree (https://www.ncbi.nlm.nih.gov/Taxonomy/CommonTree/wwwcmt.cgi). The NRC clade tree was constructed using the same methods as the extracted sequences from the highlighted tree region. Metadata and sequences used are available as Dataset EV1.

## Author contributions

**Mauricio P Contreras:** Conceptualization; data curation; investigation; visualization; writing – original draft; project administration; writing – review and editing. **Daniel Lüdke:** Conceptualization; visualization; writing – original draft; writing – review and editing. **Hsuan Pai:** Visualization. **AmirAli Toghani:** Data curation; formal analysis; visualization. **Sophien Kamoun:** Conceptualization; supervision; funding acquisition; visualization; project administration; writing – review and editing.

## Disclosure and competing interests statement

SK receives funding from industry for NLR biology and cofounded a start‐up company (Resurrect Bio Ltd.) on resurrecting disease resistance. MPC and SK have filed patents on NLR biology. MPC has received fees from Resurrect Bio Ltd.

## Supporting information



Dataset EV1
